# Universal recovery map for approximate Markov chains

**DOI:** 10.1098/rspa.2015.0623

**Published:** 2016-02

**Authors:** David Sutter, Omar Fawzi, Renato Renner

**Affiliations:** 1 Institute for Theoretical Physics, ETH Zurich, Zurich, Switzerland; 2 Department of Computing and Mathematical Sciences, Caltech, Pasadena, CA, USA; 3 LIP, ENS de Lyon, Lyon, France

**Keywords:** conditional mutual information, quantum Markov chains, recoverability, strong subadditivity

## Abstract

A central question in quantum information theory is to determine how well lost information can be reconstructed. Crucially, the corresponding recovery operation should perform well without knowing the information to be reconstructed. In this work, we show that the *quantum conditional mutual information* measures the performance of such recovery operations. More precisely, we prove that the conditional mutual information *I*(*A*:*C*|*B*) of a tripartite quantum state *ρ*_*ABC*_ can be bounded from below by its distance to the closest recovered state $R_{B\to BC}(\rho_{AB})$, where the *C*-part is reconstructed from the *B*-part only and the recovery map $R_{B\to BC}$ merely depends on *ρ*_*BC*_. One particular application of this result implies the equivalence between two different approaches to define topological order in quantum systems.

## 1. Introduction

A state *ρ*_*ABC*_ on a tripartite quantum system *A*⊗*B*⊗*C* forms a *(quantum) Markov chain* if it can be recovered from its marginal *ρ*_*AB*_ on *A*⊗*B* by a quantum operation $R_{B\to BC}$ from *B* to *B*⊗*C*, i.e.

1.1 $$\rho_{ABC}=R_{B\to BC}(\rho_{AB}).$$

An equivalent characterization of *ρ*_*ABC*_ being a quantum Markov chain is that the *conditional mutual information* *I*(*A*: *C*|*B*)_*ρ* :=_ *H*(*AB*)_*ρ*_ + *H*(*BC*)_*ρ*_ − *H*(*B*)_*ρ*_ − *H*(*ABC*)_*ρ*_ is zero [1,2], where $H{\left(A\right)}_{\rho }:=-\mathrm{tr}(\rho_{A}\log_{2}\rho_{A})$ is the von Neumann entropy. The structure of these states has been studied in various works. In particular, it has been shown that *A* and *C* can be viewed as independent conditioned on *B*, for a meaningful notion of conditioning [3].

Very recently, it has been shown that Markov states can be alternatively characterized by having a generalized Rényi conditional mutual information that vanishes [4].

A natural question that is relevant for applications is whether the above statements are robust. (In [5] an example is discussed that illustrates why this question is relevant. In [6] further explanations are given to emphasize the importance of this problem.) Specifically, one would like to have a characterization of the states that have a small (but not necessarily vanishing) conditional mutual information, i.e. *I*(*A*:*C*|*B*)≤*ε* for *ε*>0. First results revealed that such states can have a large distance to Markov chains that is independent of *ε* [7,8], which has been taken as an indication that their characterization may be difficult. However, it has subsequently been realized that a more appropriate measure instead of the distance to a (perfect) Markov chain is to consider how well (1.1) is satisfied [9,5,10,11]. This motivated the definition of *approximate Markov chains* as states where (1.1) approximately holds.

In recent work [6], it has been shown that the set of approximate Markov chains indeed coincides with the set of states with small conditional mutual information. In particular, the distance between the two terms in (1.1), which may be measured in terms of their fidelity *F*, is bounded by the conditional mutual information.^1^ More precisely, for any state *ρ*_*ABC*_ there exists a trace-preserving completely positive map $R_{B\to BC}$ (the *recovery map*) such that

1.2 $$I{\left(A:C|B\right)}_{\rho }\geq -2\log_{2}F(\rho_{ABC},R_{B\to BC}(\rho_{AB})).$$

Furthermore, a converse inequality of the form $I{\left(A:C|B\right)}_{\rho }^{2}\leq -c^{2}\log_{2}F(\rho_{ABC},R_{B\to BC}(\rho_{AB}))$, where *c* depends logarithmically on the dimension of *A* can be shown to hold [11,6].

We also note that the fidelity term in (1.2), maximized over all recovery maps, i.e.

1.3 $$F{\left(A;C|B\right)}_{\rho }:=\underset{R_{B\to BC}}{\sup}F(\rho_{ABC},R_{B\to BC}(\rho_{AB}))$$

is called *fidelity of recovery*^2^ and has been introduced and studied in [14,15]. With this quantity, the main result of [6] can be written as

1.4 $$I{\left(A:C|B\right)}_{\rho }\geq -2\log_{2}F{\left(A;C|B\right)}_{\rho }.$$

The fidelity of recovery has several natural properties, e.g. it is monotonous under local operations on *A* and *C*, and it is multiplicative [15].

The result of [6] has been extended in various ways. Based on quantum state redistribution protocols, it has been shown in [16] that (1.2) still holds if the fidelity term is replaced by the *measured relative entropy* $D_{M}(\cdot ,\cdot )$, which is generally larger, i.e. there exists a recovery map $R_{B\to BC}$ such that

1.5 $$I{\left(A:C|B\right)}_{\rho }\geq D_{M}(\rho_{ABC}∥R_{B\to BC}(\rho_{AB}))\geq -2\log_{2}F(\rho_{ABC},R_{B\to BC}(\rho_{AB})).$$

The measured relative entropy is defined as the supremum of the relative entropy with measured inputs over all projective measurements^3^ $M=\{M_{x}\}$, i.e.

1.6 $$D_{M}(\rho ∥\sigma ):=\sup\left\{D(M(\rho )∥M(\sigma )):M(\rho )=\sum_{x}\mathrm{tr}(\rho M_{x})|x\rangle \langle x|\text{ with }\sum_{x}M_{x}=\mathrm{id}\right\},$$

where {|*x*〉} is a finite set of orthonormal vectors. This quantity was studied in [17,18].

Furthermore, in [15] an alternative proof of (1.2) has been derived that uses properties of the fidelity of recovery (in particular, multiplicativity). Another recent work [19] showed how to generalize ideas from [6] to prove a remainder term for the monotonicity of the relative entropy in terms of a recovery map that satisfies (1.2).

All known proofs of (1.2) are non-constructive, in the sense that the recovery map $R_{B\to BC}$ is not given explicitly. It is merely known [6] that if *A*, *B* and *C* are finite-dimensional then $R_{B\to BC}$ can always be chosen such that it has the form

1.7 $$X_{B}\mapsto V_{BC}\rho_{BC}^{1/2}\left(\rho_{B}^{-1/2}U_{B}X_{B}U_{B}^{†}\rho_{B}^{-1/2}⊗{\mathrm{id}}_{C}\right)\rho_{BC}^{1/2}V_{BC}^{†}$$

on the support of *ρ*_*B*_, where *U*_*B*_ and *V*_*BC*_ are unitaries on *B* and *B*⊗*C*, respectively. It would be natural to expect that the choice of the recovery map that satisfies (1.2) only depends on *ρ*_*BC*_, however this is only known in special cases. One such special case is Markov chains *ρ*_*ABC*_, i.e. states for which (1.1) holds perfectly. Here a map of the form (1.7) with *V*_*BC*_=id_*BC*_ and *U*_*B*_=id_*B*_ (sometimes referred to as *transpose map* or *Petz recovery map*) serves as a perfect recovery map [1,2]. Another case where a recovery map that only depends on *ρ*_*BC*_ is known explicitly are states with a classical *B* system, i.e. qcq-states of the form $\rho_{ABC}=\sum_{b}P_{B}(b)|b\rangle \langle b|⊗\rho_{AC,b}$, where *P*_*B*_ is a probability distribution, {|*b*〉}_*b*_ an orthonormal basis on *B* and {*ρ*_*AC*,*b*_}_*b*_ a family of states on *A*⊗*C*. As discussed in [6], for such states (1.2) holds for the recovery map defined by $R_{B\to BC}(|b\rangle \langle b|)=|b\rangle \langle b|⊗\rho_{C,b}$ for all *b*, where *ρ*_*C*,*b*_=*tr*_*A*_(*ρ*_*AC*,*b*_). For general states, however, the previous results left open the possibility that the recovery map $R_{B\to BC}$ depends on the *full state* *ρ*_*ABC*_ rather than the marginal *ρ*_*BC*_ only. In particular, the unitaries *U*_*B*_ and *V*_*BC*_ in (1.7), although acting only on *B* respectively *B*⊗*C*, could have such a dependence.

In this work, we show that for any state *ρ*_*BC*_ on *B*⊗*C* there exists a recovery map $R_{B\to BC}$ that is *universal*—in the sense that the distance between *any* extension *ρ*_*ABC*_ of *ρ*_*BC*_ and $R_{B\to BC}(\rho_{AB})$ is bounded from above by the conditional mutual information *I*(*A*:*C*|*B*)_*ρ*_. In other words, we show that (1.2) remains valid if the recovery map is chosen depending on *ρ*_*BC*_ only, rather than on *ρ*_*ABC*_. This result implies a close connection between two different approaches to define topological order of quantum systems.

## 2. Main result

**Theorem 2.1**

*For any density operator ρ*_*BC*_ *on B⊗C, there exists a trace-preserving completely positive map* $R_{B\to BC}$ *such that for any extension ρ*_*ABC*_ *on A⊗B⊗C
*

2.1 $$I{\left(A:C|B\right)}_{\rho }\geq -2\log_{2}F(\rho_{ABC},R_{B\to BC}(\rho_{AB})),$$

*where A, B and C are separable Hilbert spaces.*

**Remark 2.2**

If *B* and *C* are finite-dimensional Hilbert spaces, the statement of theorem 2.1 can be tightened to

2.2 $$I{\left(A:C|B\right)}_{\rho }\geq D_{M}(\rho_{ABC}\;∥\;R_{B\to BC}(\rho_{AB})).$$

**Remark 2.3**

The recovery map $R_{B\to BC}$ predicted by theorem 2.1 has the property that it maps *ρ*_*B*_ to *ρ*_*BC*_. To see this, note that $I{\left(A:C|B\right)}_{\tilde{\rho }}=0$ for any density operator of the form ${\tilde{\rho }}_{ABC}=\rho_{A}⊗\rho_{BC}$. Theorem 2.1 thus asserts that ${\tilde{\rho }}_{ABC}$ must be equal to $R_{B\to BC}({\tilde{\rho }}_{AB})$, which implies that $\rho_{BC}\;=\;R_{B\to BC}(\rho_{B})$. We note that so far it was unknown whether recovery maps that satisfy (1.2) and have this property do exist.

We note that theorem 2.1 does not reveal any information about the structure of the recovery map that satisfies (2.1). However, if we consider a linearized version of the bound (2.1), we can make more specific statements.

**Corollary 2.4**

*For any density operator ρ*_*BC*_ *on B*⊗*C, there exists a trace-preserving completely positive map* $R_{B\to BC}$ *such that for any extension ρ*_*ABC*_ *on A*⊗*B*⊗*C*

2.3 $$I{\left(A:C|B\right)}_{\rho }\geq \frac{2}{\ln(2)}(1-F(\rho_{ABC},R_{B\to BC}(\rho_{AB})))$$

*where A, B and C are separable Hilbert spaces. Furthermore, if B and C are finite-dimensional then* $R_{B\to BC}$ *has the form*

2.4 $$X_{B}\mapsto \rho_{BC}^{1/2}U_{BC\to BC}(\rho_{B}^{-1/2}X_{B}\rho_{B}^{-1/2}⊗{\mathrm{id}}_{C})\rho_{BC}^{1/2}$$

*on the support of ρ*_*B*_, *where* $U_{BC\to BC}$ *is a unital trace-preserving map from B*⊗*C* to *B*⊗*C*.

**Remark 2.5**

Following the proof of corollary 2.4, we can deduce a more specific structure of the universal recovery map. In the finite-dimensional case, the map $R_{B\to BC}$ satisfying (2.3) can be assumed to have the form

2.5 $$X_{B}\mapsto \int V_{BC}^{s}\rho_{BC}^{1/2}(\rho_{B}^{-1/2}U_{B}^{s}X_{B}U_{B}^{s†}\rho_{B}^{-1/2}⊗{\mathrm{id}}_{C})\rho_{BC}^{1/2}V_{BC}^{s†}\mu (ds),$$

where *μ* is a probability measure on some set S, ${\left\{V_{BC}^{s}\right\}}_{s\in S}$ is a family of unitaries on *B*⊗*C* that commute with *ρ*_*BC*_, and ${\left\{U_{B}^{s}\right\}}_{s\in S}$ is a family of unitaries on *B* that commute with *ρ*_*B*_. However, the representation of the recovery map given in (2.4) has certain advantages compared to the representation (2.5). The fidelity maximized over all recovery maps of the form (2.4) can be phrased as a semidefinite programme and therefore be computed efficiently, whereas it is unknown whether the same is possible for (2.5).

We note that for almost all density operators *ρ*_*BC*_, i.e. for all *ρ*_*BC*_ except for a set of measure zero, we can replace the unitaries $U_{B}^{s}$ and $V_{BC}^{s}$ by complex matrix exponentials of the form $\rho_{B}^{it}$ and $\rho_{BC}^{it}$, respectively, with t∈R. This shows that (2.5) without the integral (the integration in (2.5) is only necessary to guarantee that the recovery map is universal) coincides with the recovery map found in [20].^4^

**Example 2.6**

For density operators with a marginal on *B*⊗*C* of the form *ρ*_*BC*_=*ρ*_*B*_⊗*ρ*_*C*_, a universal recovery map that satisfies (2.2) is uniquely defined on the support of *ρ*_*B*_—it is the transpose map, which in this case simplifies to $R_{B\to BC}:X_{B}\mapsto X_{B}⊗\rho_{C}$. It is straightforward to see that (2.2) holds. In fact, we even have equality if we consider the relative entropy (which is in general larger than the measured relative entropy), i.e.

2.6 $$I{\left(A:C|B\right)}_{\rho }=D(\rho_{ABC}∥R_{B\to BC}(\rho_{AB})).$$

The uniqueness of $R_{B\to BC}$ on the support of *ρ*_*B*_ follows by using the fact that the universal recovery map should perfectly recover the Markov state *ρ*_*AB*_⊗*ρ*_*C*_ where *ρ*_*AB*_ is a purification of *ρ*_*B*_. This forces $R_{B\to BC}$ to agree with the transpose map on the support of *ρ*_*B*_ [1,2].

The proof of theorem 2.1 is structured into two parts. We first prove the statement for finite-dimensional Hilbert spaces *B*, and *C* in §4 and then show that this implies the statement for general separable Hilbert spaces in §5. The proof of corollary 2.4 is given in §6.

## 3. Applications

A celebrated result known as *strong subadditivity* states that the conditional quantum mutual information of any density operator is non-negative [23,24], i.e.

3.1 $$I{\left(A:C|B\right)}_{\rho }\geq 0,$$

for any density operator *ρ*_*ABC*_ on *A*⊗*B*⊗*C*. Theorem 2.1 implies a strengthened version of this inequality with a remainder term that is universal in the sense that it only depends on *ρ*_*BC*_. The conditional quantum mutual information is a useful tool in different areas of physics and computer science. It is helpful to characterize measures of entanglement [6,25], analyse the correlations of quantum many-body systems [26,5], prove quantum de Finetti results [27,28] and make statements about quantum information complexity [29,30,31]. It is expected that oftentimes when (1.2) can be used, its universal version (predicted by theorem 2.1) is even more helpful.

In the following, we sketch an application where the universality result is indispensable. Theorem 2.1 can be applied to establish a connection between two alternative definitions of *topological order of quantum systems* (denoted by TQO and TQO′). Consider an *n*-spin system with n∈N. While the following statements should be understood asymptotically (in the limit n→∞), we omit the dependence on *n* in our notation for simplicity.

According to [32], a family of states ${\left\{\rho^{i}\right\}}_{i\in I}$ with $\rho^{i}\in E$ for all i∈I and |I|<∞ where E denotes a collection of states, exhibits topological quantum order (TQO) if and only if any two members of the family:

(i) are (asymptotically) orthogonal, i.e. *F*(*ρ*^*i*^,*ρ*^*j*^)=0 for all i≠j∈I and
(ii) have (asymptotically) the same marginals on any sufficiently small subregion, i.e. *tr*_*G*_*ρ*^*i*^=*tr*_*G*_*ρ*^*j*^ for all i,j∈I and *G* sufficiently large.^5^

**Figure 1.**
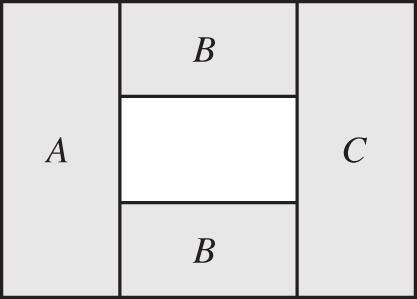
Relevant topology of the subsystems *A*, *B* and *C* such that a state *ρ*_*ABC*_ exhibits TQO′ if *I*(*A*:*C*|*B*)_*ρ*_=2*γ*>0.

Alternatively, for three regions *A*, *B* and *C* that form a certain topology F (see figure 1 and [33]), a state *ρ*_*ABC*_ on such a subspace exhibits topological quantum order (TQO′) if *I*(*A*:*C*|*B*)_*ρ*_=2*γ*>0, where *γ* denotes a *topological entanglement entropy* [33].^6^ (See [33] for more explanations on how the topological entanglement entropy is defined for the topology F depicted in figure 1.)

It is an open problem to find out how these two characterizations are related, e.g. if a family K of states on F that exhibits TQO implies that most of its members have TQO′. This connection follows by theorem 2.1. Suppose ${\left\{\rho^{i}\right\}}_{i\in I}$ with $\rho^{i}\in K$ for all i∈I shows TQO. Then consider subsystems *A*, *B* and *C* that together form a non-contractible loop. By definition of TQO, the density operators ${\left\{\rho^{i}\right\}}_{i\in I}$ share (asymptotically) the same marginals on *B*⊗*C*. Applying theorem 2.1 to this common marginal, together with the continuity of the conditional mutual information [35] ensures that there exist a recovery map $R_{B\to BC}$ such that for any i∈I,

3.2 $$I{\left(A:C|B\right)}_{\rho^{i}}\geq -2\log F(\rho_{ABC}^{i},R_{B\to BC}(\rho_{AB}^{i})).$$

Since the density operators ${\left\{\rho_{ABC}^{i}\right\}}_{i\in I}$ are (asymptotically) orthogonal, share (asymptotically) the same marginals on *A*⊗*B*, and the fidelity is continuous in its inputs [12,13], this implies that for all i∈I, except of a single element, we have

3.3 $$I{\left(A:C|B\right)}_{\rho^{i}}\geq \mathrm{const}.>0.$$

## 4. Proof for finite dimensions

Throughout this section, we assume that the Hilbert spaces *B* and *C* are finite-dimensional. In the proof Steps 1–3 below, we also make the same assumption for *A*, but then drop it in Step 4. We start by explaining why (2.2) is a tightened version of (2.1) which was noticed in [16]. Let *D*_*α*_(⋅∥⋅) be the *α*-*Quantum Rényi Divergence* as defined in [36,37] with $D_{1}(\rho ∥\sigma )=D(\rho ∥\sigma ):=\mathrm{tr}(\rho (\log\rho -\log\sigma ))$ and $D_{\alpha }(\rho ∥\sigma ):=(1/(\alpha -1))\log\mathrm{tr}({\left(\sigma^{(1-\alpha )/2\alpha }\rho \sigma^{(1-\alpha )/2\alpha }\right)}^{\alpha })$ for any density operator *ρ*, any non-negative operator *σ* such that supp(*ρ*)⊆supp(*σ*) and any α∈(0,1)∪(1,∞). By definition of the measured relative entropy (see (1.6)), we find for any two states *ρ* and *σ*

4.1 $$\begin{array}{rl} D_{M}(\rho ∥\sigma ) & =\underset{M\in M}{\sup}D(M(\rho )∥M(\sigma ))\geq \underset{M\in M}{\sup}D_{1/2}(M(\rho )∥M(\sigma ))\\ & =-2\log_{2}\underset{M\in M}{\inf}F(M(\rho ),M(\sigma ))=-2\log_{2}F(\rho ,\sigma ), \end{array}$$

where $M:=\{M:M(\rho )=\sum_{x}\mathrm{tr}(\rho M_{x})|x\rangle \langle x|$ with ∑ _*x*_*M*_*x*_=id} and {|*x*〉} is a family of orthonormal vectors. The inequality step uses that *α*↦*D*_*α*_(*ρ*∥*σ*) is a monotonically non-decreasing function in *α* [36], theorem 7 and the final step follows from the fact that for any two states there exists an optimal measurement that does not increase their fidelity [38], §3.3. As a result, in order to prove theorem 2.1 for finite-dimensional *B* and *C* it suffices to prove (2.2).

We first derive a proposition (proposition 4.1) and then show how it can be used to prove (2.2) (and, hence, theorem 2.1). The proposition refers to a family of functions

4.2 $$D(A⊗B⊗C)∋\rho \mapsto \Delta_{R}(\rho )\in R\cup \{-\infty \},$$

parametrized by recovery maps R∈TPCP(B,B⊗C), where TPCP(*B*,*B*⊗*C*) denotes the set of trace-preserving completely positive maps from *B* to *B*⊗*C* and *D*(*A*⊗*B*⊗*C*) denotes the set of density operators on *A*⊗*B*⊗*C*. Subsequently in the proof, the function family $\Delta_{R}(\cdot )$ will be constructed as the difference of the two terms in (2.2) (see equation (4.38)) such that $\Delta_{R}(\rho )\geq 0$ corresponds to (2.2). The proposition asserts that if for any extension *ρ*_*ABC*_ of *ρ*_*BC*_ we have $\Delta_{R}(\rho )\geq 0$ for some R∈TPCP(B,B⊗C) and provided the function family $\Delta_{R}(\cdot )$ satisfies certain properties described below, then there exists a single recovery map R for which $\Delta_{R}(\rho )\geq 0$ for all extensions *ρ*_*ABC*_ of *ρ*_*BC*_ on a fixed *A* system. We note that the precise form of the function family $\Delta_{R}(\cdot )$ is irrelevant for proposition 4.1 as long as it satisfies a list of properties as stated below.

As described above, our goal is to prove that there exists a recovery map $R_{B\to BC}$ such that $\Delta_{R}(\rho )\geq 0$ for all *ρ*_*ABC*_∈*D*(*A*⊗*B*⊗*C*) with a fixed marginal *ρ*_*BC*_ on *B*⊗*C*. To formulate our argument more concisely, we introduce some notation. For any set S of density operators *ρ*_*ABC*_∈*D*(*A*⊗*B*⊗*C*), we define

4.3 $$\Delta_{R}(S):=\underset{\rho \in S}{\inf}\Delta_{R}(\rho ).$$

The desired statement then reads as $\Delta_{R}(S)\geq 0$, for any set S of states on *A*⊗*B*⊗*C* with a fixed marginal *ρ*_*BC*_. Furthermore, for any fixed states $\rho_{ABC}^{0}$ and *ρ*_*ABC*_ on *A*⊗*B*⊗*C* and *p*∈[0,1], we define

4.4 $$\rho_{\hat{A}ABC}^{p}:=(1-p)|0\rangle \langle 0{|}_{\hat{A}}⊗\rho_{ABC}^{0}+p|1\rangle \langle 1{|}_{\hat{A}}⊗\rho_{ABC},$$

where $\hat{A}$ is an additional system with two orthogonal states |0〉 and |1〉. More generally, for any fixed state $\rho_{ABC}^{0}$ and for any set S of density operators *ρ*_*ABC*_ we set

4.5 $$S^{p}:=\left\{\rho_{\hat{A}ABC}^{p}:\;\rho_{ABC}\in S\right\}.$$

**Required properties of the Δ-function 1**

(i) For any $\rho_{ABC}^{0},\rho_{ABC}\in D(A⊗B⊗C)$ with identical marginals $\rho_{BC}^{0}=\rho_{BC}$ on *B*⊗*C*, for any R∈TPCP(B,B⊗C), and for any *p*∈[0,1] we have $\Delta_{R}(\rho^{p})=(1-p)\Delta_{R}(\rho^{0})+p\Delta_{R}(\rho )$.
(ii) For any $R,R^{\prime }\in \mathrm{TPCP}(B,B⊗C)$, for any *α*∈[0,1] and $\bar{R}=\alpha R+(1-\alpha )R^{\prime }$ we have $\Delta_{\bar{R}}(\rho )\geq \alpha \Delta_{R}(\rho )+(1-\alpha )\Delta_{R^{\prime }}(\rho )$ for all *ρ*∈*D*(*A*⊗*B*⊗*C*).
(iii) For any R∈TPCP(B,B⊗C), the function $D(A⊗B⊗C)∋\rho \mapsto \Delta_{R}(\rho )\in R\cup \{-\infty \}$ is upper semicontinuous.
(iv) For any *ρ*∈*D*(*A*⊗*B*⊗*C*), the function $\mathrm{TPCP}(B,B⊗C)∋R\mapsto \Delta_{R}(\rho )\in R\cup \{-\infty \}$ is upper semicontinuous.

Property (i) implies that for any state $\rho_{ABC}^{0}$, for any set S of operators *ρ*_*ABC*_ with $\rho_{BC}=\rho_{BC}^{0}$, and for any *p*∈[0,1] we have

4.6 $$\Delta_{R}(S^{p})=\underset{\rho \in S}{\inf}\Delta_{R}(\rho^{p})=(1-p)\Delta_{R}(\rho^{0})+p\underset{\rho \in S}{\inf}\Delta_{R}(\rho )=(1-p)\Delta_{R}(\rho^{0})+p\Delta_{R}(S).$$

Similarly, property (ii) implies

4.7 $$\begin{array}{rl} \Delta_{\bar{R}}(S) & =\underset{\rho \in S}{\inf}\Delta_{\bar{R}}(\rho )\geq \underset{\rho \in S}{\inf}\{\alpha \Delta_{R}(\rho )+(1-\alpha )\Delta_{R^{\prime }}(\rho )\}\\ & \geq \alpha \underset{\rho \in S}{\inf}\Delta_{R}(\rho )+(1-\alpha )\underset{\rho \in S}{\inf}\Delta_{R^{\prime }}(\rho )=\alpha \Delta_{R}(S)+(1-\alpha )\Delta_{R^{\prime }}(S). \end{array}$$

**Proposition 4.1**

*Let A,B and C be finite-dimensional Hilbert spaces*, P⊆TPCP(B,B⊗C) *be compact and convex*, S *be a set of density operators on A*⊗*B*⊗*C with identical marginals on B*⊗*C, and* $\Delta_{R}(\cdot )$ *be a family of functions of the form* (4.2) *that satisfies properties* (i)–(iv). *Then*

4.8 $$\forall \rho \in S\exists R\in P:\Delta_{R}(\rho )\geq 0\;⟹\;\exists \bar{R}\in P:\Delta_{\bar{R}}(S)\geq 0.$$

We now proceed in four steps. In the first, we prove proposition 4.1 for finite sets S. This is done by induction over the cardinality of the set S. We show that if the statement of proposition 4.1 is true for all sets S with |S|=n, this implies that it remains valid for all sets S with |S|=n+1. In Step 2, we use an approximation step to extend this to infinite sets S which then completes the proof of proposition 4.1. In the final two steps, we show how to conclude the statement of theorem 2.1 for the finite-dimensional case from that. In Step 3, we prove (2.2) for the case where the recovery map that satisfies (2.2) could still depend on the dimension of the system *A*. In Step 4, we show how this dependency can be removed.

Proposition 4.1 resembles Sion’s minimax theorem [39]. After the completion of this work, it has been noticed that the argument done by proposition 4.1 in this work can be alternatively carried out using Sion’s minimax theorem (see [40] for a detailed explanation).

### (a) Step 1. Proof of proposition thm4.1 for finite size sets S

We proceed by induction over the cardinality n:=|S| of the set S of density operators. More precisely, the induction hypothesis is that for any finite-dimensional Hilbert space *A* and any set S of size *n* consisting of density operators on *A*⊗*B*⊗*C* with fixed marginal *ρ*_*BC*_ on *B*⊗*C*, the statement (4.8) holds. For *n*=1, this hypothesis holds trivially for $\bar{R}=R$.^7^

We now prove the induction step. Suppose that the induction hypothesis holds for some *n*. Let *A* be a finite-dimensional Hilbert space and let $S\cup \{\rho_{ABC}^{0}\}$ be a set of cardinality *n*+1 where S is a set of states on *A*⊗*B*⊗*C* with fixed marginal *ρ*_*BC*_ on *B*⊗*C* of cardinality *n* and $\rho_{ABC}^{0}$ is another state with $\rho_{BC}^{0}=\rho_{BC}$. We need to prove that there exists a recovery map ${\bar{R}}_{B\to BC}\in P$ such that

4.9 $$\Delta_{\bar{R}}(S\cup \{\rho_{ABC}^{0}\})\geq 0.$$

Let *p*∈[0,1] and consider the set $S^{p}$ as defined in (4.5). In the following, we view the states *ρ*^*p*^ (see equation (4.4)) in this set as tripartite states on $(\hat{A}⊗A)⊗B⊗C$, i.e. we regard the system $\hat{A}⊗A$ as one (larger) system. The induction hypothesis applied to the extension space $\hat{A}⊗A$ and the set $S^{p}$ (of size *n*) of states on $(\hat{A}⊗A)⊗B⊗C$ implies the existence of a map $R_{B\to BC}^{p}\in P$ such that

4.10 $$\Delta_{R^{p}}(S^{p})\geq 0.$$

As by assumption the function $D(A⊗B⊗C)∋\rho \mapsto \Delta_{R^{p}}(\rho )\in R\cup \{-\infty \}$ satisfies property (i) (and hence also equation (4.6)), we obtain

4.11 $$(1-p)\Delta_{R^{p}}(\rho^{0})+p\Delta_{R^{p}}(S)\geq 0.$$

This implies that

4.12 $$\Delta_{R^{p}}(\rho^{0})\geq 0\;\mathrm{or}\;\Delta_{R^{p}}(S)\geq 0.$$

Furthermore, for *p*=0 the left inequality holds and for *p*=1 the right inequality holds. By choosing $K_{0}=\{p\in [0,1]:\Delta_{R^{p}}(\rho^{0})\geq 0\}$ and $K_{1}=\{p\in [0,1]:\Delta_{R^{p}}(S)\geq 0\}$, the *touching sets lemma* (see lemma 11.1 in the electronic supplementary material) implies that for any *δ*>0 there exist *u*,*v*∈[0,1] with 0≤*v*−*u*≤*δ* such that

4.13 $$\Delta_{R^{u}}(\rho^{0})\geq 0\;\mathrm{and}\;\Delta_{R^{v}}(S)\geq 0.$$

Note also that $R_{B\to BC}^{u}$, $R_{B\to BC}^{v}\in P$, as by the induction hypothesis $R_{B\to BC}^{p}\in P$ for any *p*∈[0,1].

We will use this to prove that the recovery map $\tilde{R}\in P$ defined by

4.14 $$\tilde{R}:=\alpha R^{u}+(1-\alpha )R^{v},$$

for an appropriately chosen *α*∈[0,1], satisfies

4.15 $$\Delta_{\tilde{R}}(\rho^{0})\geq -c\delta \;\mathrm{and}\;\Delta_{\tilde{R}}(S)\geq -c\delta ,$$

where *c* is a constant defined by

4.16 $$c:=4\max_{R\in \mathrm{TPCP}(B,B⊗C)}\max_{\rho \in D(A⊗B⊗C)}\Delta_{R}(\rho )<\infty .$$

Properties (iii) and (iv) together with simple topological facts about the set of density operators and the set of trace-preserving completely positive maps (see lemma 10.1 and remark 10.3 stated in the electronic supplementary material) ensure that the two maxima in (4.16) are attained which implies by the definition of the codomain of $\Delta_{R}(\cdot )$ (see equation (4.2)) that *c* is finite. In other words, for any *δ*>0 there exists a recovery map ${\tilde{R}}^{\delta }\in P$ such that

4.17 $$\Delta_{{\tilde{R}}^{\delta }}(S\cup \{\rho^{0}\})\geq -c\delta .$$

The compactness of P ensures that there exists a recovery map $\bar{R}\in P$ and a sequence ${\left\{\delta_{n}\right\}}_{n\in N}$ such that

4.18 $$\lim_{n\to \infty }\delta_{n}=0\;\mathrm{and}\;\lim_{n\to \infty }{\tilde{R}}^{\delta_{n}}=\bar{R}.$$

Because of (4.17) we have

4.19 $$\underset{n\to \infty }{lim sup}\Delta_{{\tilde{R}}^{\delta_{n}}}(S\cup \{\rho^{0}\})\geq \lim_{n\to \infty }-c\delta_{n}=0,$$

which together with property (iv) implies that

4.20 $$\begin{array}{rl} \Delta_{\bar{R}}(S\cup \{\rho^{0}\}) & =\min_{\rho \in S\cup \{\rho^{0}\}}\Delta_{\bar{R}}(\rho )\geq \min_{\rho \in S\cup \{\rho^{0}\}}\underset{n\to \infty }{lim sup}\Delta_{{\tilde{R}}^{\delta_{n}}}(\rho )\geq \underset{n\to \infty }{lim sup}\min_{\rho \in S\cup \{\rho^{0}\}}\Delta_{{\tilde{R}}^{\delta_{n}}}(\rho )\\ & =\underset{n\to \infty }{lim sup}\Delta_{{\tilde{R}}^{\delta_{n}}}(S\cup \{\rho^{0}\})\geq 0, \end{array}$$

and thus proves (4.9).

It thus remains to show (4.15). To simplify the notation, let us define

4.21 $$\Lambda^{0}:=\Delta_{R^{u}}(\rho^{0})\;\mathrm{and}\;\Lambda^{1}:=\Delta_{R^{v}}(S)$$

as well as

4.22 $${\bar{\Lambda }}^{0}:=\Delta_{R^{v}}(\rho^{0})\;\mathrm{and}\;{\bar{\Lambda }}^{1}:=\Delta_{R^{u}}(S).$$

It follows from (4.11) that

4.23 $$(1-u)\Lambda^{0}+u{\bar{\Lambda }}^{1}\geq 0.$$

Similarly, we have

4.24 $$(1-v){\bar{\Lambda }}^{0}+v\Lambda^{1}\geq 0.$$

As by assumption the function $\Delta_{R}(\cdot )$ satisfies property (ii) we find together with (4.24) that for any *α*∈[0,1] and $\bar{R}=\alpha R^{u}+(1-\alpha )R^{v}$,

4.25 $$\Delta_{\bar{R}}(\rho^{0})\geq \alpha \Delta_{R^{u}}(\rho^{0})+(1-\alpha )\Delta_{R^{v}}(\rho^{0})=\alpha \Lambda^{0}+(1-\alpha ){\bar{\Lambda }}^{0}\geq \alpha \Lambda^{0}-(1-\alpha )\frac{v}{1-v}\Lambda^{1}.$$

(If *v*=1 it suffices to consider the case *α*=1 so that the last term can be omitted; cf. equation (4.29).) Analogously, using (4.7) and (4.23), we find

4.26 $$\Delta_{\bar{R}}(S)\geq \alpha \Delta_{R^{u}}(S)+(1-\alpha )\Delta_{R^{v}}(S)=\alpha {\bar{\Lambda }}^{1}+(1-\alpha )\Lambda^{1}\geq -\alpha \frac{1-u}{u}\Lambda^{0}+(1-\alpha )\Lambda^{1}.$$

(If *u*=0 it suffices to consider the case *α*=0; cf. equation (4.32).)

To conclude the proof of (4.15), it suffices to choose *α*∈[0,1] such that the terms on the right-hand side of (4.25) and (4.26) satisfy

4.27 $$\alpha \Lambda^{0}-(1-\alpha )\frac{v}{1-v}\Lambda^{1}\geq -c\delta$$

and

4.28 $$-\alpha \frac{1-u}{u}\Lambda^{0}+(1-\alpha )\Lambda^{1}\geq -c\delta .$$

Let us first assume that $u\geq \frac{1}{2}$. Since *Λ*^0^ and *Λ*^1^ are non-negative (see equation (4.13)), we may choose *α*∈[0,1] such that

4.29 $$\alpha (1-v)\Lambda^{0}=(1-\alpha )v\Lambda^{1}.$$

This immediately implies that the left-hand side of (4.27) equals 0, so that the inequality holds. As $\frac{1}{2}\leq u\leq v\leq 1$ and *v*−*u*≤*δ* we have

4.30 $$\left|\frac{1-u}{u}-\frac{1-v}{v}\right|\leq 4\delta .$$

Combining this with (4.29), we find

4.31 $$-\alpha \frac{1-u}{u}\Lambda^{0}+(1-\alpha )\Lambda^{1}\geq -\alpha \Lambda^{0}\left(\frac{1-v}{v}+4\delta \right)+(1-\alpha )\Lambda^{1}=-4\alpha \Lambda^{0}\delta \geq -4\Lambda^{0}\delta ,$$

which proves (4.28) because by (4.16) we have *Λ*^0^≤*c*/4.

Analogously, if $u<\frac{1}{2}$, choose *α*∈[0,1] such that

4.32 $$\alpha (1-u)\Lambda^{0}=(1-\alpha )u\Lambda^{1}.$$

This immediately implies that the left-hand side of (4.28) equals 0, so that the inequality holds. Furthermore, for *δ*>0 sufficiently small such that $v\leq \frac{1}{2}$, we obtain

4.33 $$\left|\frac{v}{1-v}-\frac{u}{1-u}\right|<4\delta .$$

Together with (4.32) this implies

4.34 $$\alpha \Lambda^{0}-(1-\alpha )\frac{v}{1-v}\Lambda^{1}\geq \alpha \Lambda^{0}-(1-\alpha )\Lambda^{1}\left(\frac{u}{1-u}+4\delta \right)=-4(1-\alpha )\Lambda^{1}\delta \geq -4\Lambda^{1}\delta ,$$

which establishes (4.27). This concludes the proof of proposition 4.1 for sets S of finite size.

### (b) Step 2. Extension to infinite sets S

All that remains to be done to prove proposition 4.1 is to generalize the statement to arbitrarily large sets S. In fact, we show that there exists a recovery map $R_{B\to BC}\in P$ such that $\Delta_{R}(S)\geq 0$, where S is the set of all density operators on *A*⊗*B*⊗*C* for a fixed finite-dimensional Hilbert space *A* and a fixed marginal *ρ*_*BC*_.

Note first that this set S of all density operators on *A*⊗*B*⊗*C* with fixed marginal *ρ*_*BC*_ on *B*⊗*C* is compact (see lemma 10.2 in the electronic supplementary material). This implies that for any *ε*>0 there exists a finite set $S^{\varepsilon }$ of density operators on *A*⊗*B*⊗*C* such that any ρ∈S is *ε*-close to an element of $S^{\varepsilon }$. We further assume without loss of generality that $S^{\varepsilon^{\prime }}\subset S^{\varepsilon }$ for *ε*′≥*ε*. Let $R^{\varepsilon }\in \mathrm{TPCP}(B,B⊗C)$ be a map such that $\Delta_{R^{\varepsilon }}(S^{\varepsilon })\geq 0$, whose existence follows from the validity of proposition 4.1 for sets of finite size (which we proved in Step 1). Since the set TPCP(*B*,*B*⊗*C*) is compact (see remark 10.3 in the electronic supplementary material) there exists a decreasing sequence ${\left\{\varepsilon_{n}\right\}}_{n\in N}$ and $\bar{R}\in \mathrm{TPCP}(B,B⊗C)$ such that

4.35 $$\lim_{n\to \infty }\varepsilon_{n}=0\;\mathrm{and}\;\bar{R}=\lim_{n\to \infty }R^{\varepsilon_{n}}.$$

Combining this with property (iv) gives for all n∈N

4.36 $$\begin{array}{rl} \Delta_{\bar{R}}(S^{\varepsilon_{n}}) & =\underset{\rho \in S^{\varepsilon_{n}}}{\inf}\Delta_{\bar{R}}(\rho )\geq \underset{\rho \in S^{\varepsilon_{n}}}{\inf}\underset{m\to \infty }{lim sup}\Delta_{{\bar{R}}^{\varepsilon_{m}}}(\rho )\geq \underset{m\to \infty }{lim sup}\underset{\rho \in S^{\varepsilon_{n}}}{\inf}\Delta_{{\bar{R}}^{\varepsilon_{m}}}(\rho )\\ & \geq \underset{m\to \infty }{lim sup}\underset{\rho \in S^{\varepsilon_{m}}}{\inf}\Delta_{{\bar{R}}^{\varepsilon_{m}}}(\rho )=\underset{m\to \infty }{lim sup}\Delta_{{\bar{R}}^{\varepsilon_{m}}}(S^{\varepsilon_{m}})\geq 0, \end{array}$$

where the third inequality holds since $S^{\varepsilon_{n}}\subset S^{\varepsilon_{m}}$ for *ε*_*n*_≥*ε*_*m*_, respectively *n*≤*m*. The final inequality follows from the defining property of $R^{\varepsilon }$. For any fixed ρ∈S and for all n∈N, let $\rho^{n}\in S^{\varepsilon_{n}}$ be such that $\lim_{n\to \infty }\rho^{n}=\rho \in S$. (By definition of $S^{\varepsilon_{n}}$ it follows that such a sequence ${\left\{\rho^{n}\right\}}_{n\in N}$ with $\rho^{n}\in S^{\varepsilon_{n}}$ always exists.) Property (iii) together with (4.36) yields

4.37 $$\Delta_{\bar{R}}(\rho )=\Delta_{\bar{R}}\left(\lim_{n\to \infty }\rho^{n}\right)\geq \underset{n\to \infty }{lim sup}\Delta_{\bar{R}}(\rho^{n})\geq \underset{n\to \infty }{lim sup}\Delta_{\bar{R}}(S^{\varepsilon_{n}})\geq 0.$$

Since (4.37) holds for any ρ∈S, we obtain $\Delta_{\bar{R}}(S)\geq 0$, which completes the proof of proposition 4.1.

### (c) Step 3. From proposition thm4.1 to theorem thm2.1 for fixed system *A*

We next show that theorem 2.1, for the case where *A* is a fixed finite-dimensional system, follows from proposition 4.1. For this we use proposition 4.1 for the function family

4.38 $$\begin{array}{rl} & \Delta_{R}:D(A⊗B⊗C)\to R\cup \{-\infty \}\\ & \;\rho_{ABC}\mapsto I{\left(A:C|B\right)}_{\rho }-D_{M}(\rho_{ABC},R_{B\to BC}(\rho_{AB})), \end{array}$$

with $R_{B\to BC}\in \mathrm{TPCP}(B,B⊗C)$. We note that since *C* is finite-dimensional this implies that $\Delta_{R}(\rho )<\infty$ for all *ρ*∈*D*(*A*⊗*B*⊗*C*). To apply proposition 4.1, we have to verify that the function family $D(A⊗B⊗C)∋\rho \mapsto \Delta_{R}(\rho )\in R\cup \{-\infty \}$ of the form (4.38) satisfies the assumptions of the proposition. This is ensured by the following lemma.

**Lemma 4.2**

*Let A be a separable and B and C finite-dimensional Hilbert spaces. The function family* $\Delta_{R}(\cdot )$ *defined by* (*4.38*) *satisfies properties* (i)–(iv).

**Proof.**

We first verify that the function $\Delta_{R}(\cdot )$ satisfies property (i). For any state *ρ*^*p*^ of the form (4.4), we have by the definition of the mutual information

4.39 $$I{\left(\hat{A}A:C|B\right)}_{\rho^{p}}=H{\left(C|B\right)}_{\rho^{p}}-H{\left(C|BA\hat{A}\right)}_{\rho^{p}}.$$

Because $\rho_{BC}^{0}=\rho_{BC}$, the first term, *H*(*C*|*B*)_*ρ*^*p*^_, is independent of *p*, i.e. *H*(*C*|*B*)_*ρ*^*p*^_=*H*(*C*|*B*)_*ρ*^0^_=*H*(*C*|*B*)_*ρ*_. The second term can be written as an expectation over $\hat{A}$, i.e.

4.40 $$H{\left(C|BA\hat{A}\right)}_{\rho^{p}}=\left(1-p\right)H{\left(C|BA\right)}_{\rho^{0}}+pH{\left(C|BA\right)}_{\rho }.$$

As a result, we find

4.41 $$I{\left(\hat{A}A:C|B\right)}_{\rho^{p}}=(1-p)I{\left(A:C|B\right)}_{\rho^{0}}+pI{\left(A:C|B\right)}_{\rho }.$$

The density operator $R_{B\to BC}(\rho_{\hat{A}AB}^{p})$ can be written as

4.42 $$R_{B\to BC}(\rho_{\hat{A}AB}^{p})=(1-p)|0\rangle \langle 0{|}_{\hat{A}}⊗R_{B\to BC}(\rho_{AB}^{0})+p|1\rangle \langle 1{|}_{\hat{A}}⊗R_{B\to BC}(\rho_{AB}).$$

We can thus apply lemma 9.3 given in the electronic supplementary material (which states a linearity property of the measured relative entropy for orthogonal states), from which we obtain

4.43 $$\begin{array}{r} D_{M}\left(\rho_{\hat{A}ABC}^{p}∥R_{B\to BC}(\rho_{\hat{A}AB}^{p})\right)=(1-p)D_{M}\left(\rho_{ABC}^{0}∥R_{B\to BC}(\rho_{AB}^{0})\right)+pD_{M}\left(\rho_{ABC}^{p}∥R_{B\to BC}(\rho_{AB}^{p})\right). \end{array}$$

Equations (4.41) and (4.43) imply that

4.44 $$\Delta_{R}(\rho^{p})=(1-p)\Delta_{R}(\rho^{0})+p\Delta_{R}(\rho ),$$

which concludes the proof of property (i).

That $\Delta_{R}(\cdot )$ satisfies property (ii) can be seen as follows. Let $R_{B\to BC},R_{B\to BC}^{\prime }\in \mathrm{TPCP}(B,B⊗C)$, *α*∈[0,1] and ${\bar{R}}_{B\to BC}=\alpha R_{B\to BC}+(1-\alpha )R_{B\to BC}^{\prime }$. Since the measured relative entropy is convex in the second argument (see lemma 9.4 given in the electronic supplementary material) we find that for any state *ρ*_*ABC*_ on *A*⊗*B*⊗*C*

4.45 $$\begin{array}{rl} & D_{M}(\rho_{ABC}∥{\bar{R}}_{B\to BC}(\rho_{AB}))=D_{M}\left(\rho_{ABC}∥\alpha R_{B\to BC}(\rho_{AB})+(1-\alpha )R_{B\to BC}^{\prime }(\rho_{AB})\right)\\ & \;\leq \alpha D_{M}(\rho_{ABC}∥R_{B\to BC}(\rho_{AB}))+(1-\alpha )D_{M}(\rho_{ABC}∥R_{B\to BC}^{\prime }(\rho_{AB})) \end{array}$$

and hence

4.46 $$\Delta_{\bar{R}}(\rho )\geq \alpha \Delta_{R}(\rho )+(1-\alpha )\Delta_{R^{\prime }}(\rho ).$$

We next verify that the function $\Delta_{R}(\cdot )$ satisfies property (iii). The Alicki–Fannes inequality ensures that $D(A⊗B⊗C)∋\rho \mapsto I{\left(A:C|B\right)}_{\rho }\in R^{+}$ is continuous since *C* is finite-dimensional [35]. By the definition of $\Delta_{R}(\cdot )$ it thus suffices to show that $D(A⊗B⊗C)∋\rho_{ABC}\mapsto D_{M}(\rho_{ABC}∥R_{B\to BC}(\rho_{AB}))\in R^{+}$ is lower semicontinuous. Let ${\left\{\rho_{ABC}^{n}\right\}}_{n\in N}$ be a sequence of states on *A*⊗*B*⊗*C* such that $\lim_{n\to \infty }\rho_{ABC}^{n}=\rho_{ABC}\in D(A⊗B⊗C)$. By definition of the measured relative entropy (see (1.6)), we find for $M:=\{M:M(\rho )=\sum_{x}\mathrm{tr}(\rho M_{x})|x\rangle \langle x|\text{ with }\sum_{x}M_{x}=\mathrm{id}\}$,

4.47 $$\begin{array}{rl} \underset{n\to \infty }{lim inf}D_{M}(\rho_{ABC}^{n}∥R_{B\to BC}(\rho_{AB}^{n})) & =\underset{n\to \infty }{lim inf}\underset{M\in M}{\sup}D(M(\rho_{ABC}^{n})∥M(R_{B\to BC}(\rho_{AB}^{n})))\\ & \geq \underset{M\in M}{\sup}\underset{n\to \infty }{lim inf}D(M(\rho_{ABC}^{n})∥M(R_{B\to BC}(\rho_{AB}^{n})))\\ & \geq \underset{M\in M}{\sup}D(M(\rho_{ABC})∥M(R_{B\to BC}(\rho_{AB})))\\ & =D_{M}(\rho_{ABC}∥R_{B\to BC}(\rho_{AB})). \end{array}$$

In the penultimate step, we use that the relative entropy is lower semicontinuous [41], Exercise 7.22 and that M as well as $R_{B\to BC}$ are linear and bounded operators and hence continuous.

We finally show that $\Delta_{R}(\cdot )$ fulfils property (iv). It suffices to verify that $\mathrm{TPCP}(B,B⊗C)∋R\mapsto D_{M}(\rho_{ABC}∥R(\rho_{AB}))\in R^{+}$ is lower semicontinuous where by definition of the measured relative entropy (see (1.6)) we have that

4.48 $$D_{M}(\rho_{ABC}∥R(\rho_{AB}))=\underset{M\in M}{\sup}D(M(\rho_{ABC})∥M(R_{B\to BC}(\rho_{AB}))).$$

Note that since R and M are linear bounded operators and hence continuous and the relative entropy for two states *σ*_1_ and *σ*_2_ is defined by $D(\sigma_{1}∥\sigma_{2}):=\mathrm{tr}(\sigma_{1}(\log\sigma_{1}-\log\sigma_{2}))$ we find that $R\mapsto D(M(\rho_{ABC})∥M(R_{B\to BC}(\rho_{AB})))$ is continuous as the logarithm $R^{+}∋x\mapsto \log x\in R$ is continuous. Since the supremum of continuous functions is lower semicontinuous [42], ch. IV, Section 6.2, Theorem 4, the assertion follows. ▪

What remains to be shown in order to apply proposition 4.1 is that for any ρ∈S where S is the set of states on *A*⊗*B*⊗*C* with a fixed marginal *ρ*_*BC*_ on *B*⊗*C*, there exists a recovery map $R_{B\to BC}\in P$ such that $\Delta_{R}(\rho )\geq 0$. By choosing P=TPCP(B,B⊗C), the main result of [16] however precisely proves this. We have thus shown that $\Delta_{R}(\rho )\geq 0$ holds for a universal recovery map $R_{B\to BC}\in P$, so that (2.2) follows for any fixed dimension of the *A* system. This proves the statement of remark 2.2 (and, hence, theorem 2.1) for the case where *A* is a fixed finite-dimensional Hilbert space.

### (d) Step 4. Independence from the *A* system

Let S be the set of all density operators on $\bar{A}⊗B⊗C$ with a fixed marginal *ρ*_*BC*_ on *B*⊗*C*, where *B* and *C* are finite-dimensional Hilbert spaces and $\bar{A}$ is the infinite-dimensional Hilbert space ℓ^2^ of square summable sequences. We now show that there exists a recovery map $R_{B\to BC}$ such that $\Delta_{R}(S)\geq 0$.

Let ${\left\{\Pi_{\bar{A}}^{a}\right\}}_{a\in N}$ be a sequence of finite-rank projectors on $\bar{A}$ that converges to ${\mathrm{id}}_{\bar{A}}$ with respect to the weak operator topology. Let $S^{a}$ denote the set of states whose marginal on $\bar{A}$ is contained in the support of $\Pi_{\bar{A}}^{a}$ and with the same fixed marginal *ρ*_*BC*_ on *B*⊗*C* as the elements of S. For all a∈N, let $R_{B\to BC}^{a}$ denote a recovery map that satisfies $\Delta_{R^{a}}(S^{a})\geq 0$. Note that the existence of such maps is already established by the proof of theorem 2.1 for the finite-dimensional case. As the set of trace-preserving completely positive maps on finite-dimensional systems is compact (see remark 10.3 in the electronic supplementary material) there exists a subsequence ${\left\{a_{i}\right\}}_{i\in N}$ such that $\lim_{i\to \infty }a_{i}=\infty$ and $\lim_{i\to \infty }R^{a_{i}}=\bar{R}\in \mathrm{TPCP}(B,B⊗C)$. For every ρ∈S, there exists a sequence of states ${\left\{\rho^{a}\right\}}_{a\in N}$ with $\rho^{a}\in S^{a}$ that converges to *ρ* in the trace norm (see lemma 12.3 in the electronic supplementary material). Lemma 4.2 (in particular properties (iii) and (iv)), yields for any ρ∈S

4.49 $$\begin{array}{rl} \Delta_{\bar{R}}(\rho ) & \geq \underset{a\to \infty }{lim sup}\Delta_{\bar{R}}(\rho^{a})\geq \underset{a\to \infty }{lim sup}\underset{i\to \infty }{lim sup}\Delta_{R^{a_{i}}}(\rho^{a})\geq \underset{a\to \infty }{lim sup}\underset{i\to \infty }{lim sup}\underset{\rho \in S^{a}}{\inf}\Delta_{R^{a_{i}}}(\rho )\\ & \geq \underset{i\to \infty }{lim sup}\underset{\rho \in S^{a_{i}}}{\inf}\Delta_{R^{a_{i}}}(\rho )=\underset{i\to \infty }{lim sup}\Delta_{R^{a_{i}}}(S^{a_{i}})\geq 0. \end{array}$$

The fourth inequality follows since *a*_*i*_≥*a* for large enough *i* and since this implies that $S^{a_{i}}⊃S^{a}$, and the final inequality follows by definition of $R^{a_{i}}$. This shows that $\Delta_{\bar{R}}(S)\geq 0$.

To retrieve the statement of remark 2.2 (and hence theorem 2.1 for finite-dimensional *B* and *C*), we need to argue that this same map $\bar{R}$ remains valid when we consider any separable space *A*. In order to do this, observe that any separable Hilbert space *A* can be isometrically embedded into $\bar{A}$ [21], Theorem II.7. To conclude, it suffices to remark that $\Delta_{\bar{R}}$ is invariant under isometries applied on the space *A*.

## 5. Extension to infinite dimensions

In this section, we show how to obtain the statement of theorem 2.1 for separable (not necessarily finite-dimensional) Hilbert spaces *A*, *B*, *C* from the finite-dimensional case that has been proven in §4. For trace non-increasing completely positive maps $R_{B\to BC}$, we define the function family

5.1 $$\begin{array}{rl} & {\bar{\Delta }}_{R}:D(A⊗B⊗C)\to R\cup \{-\infty \}\\ & \;\rho_{ABC}\mapsto F(\rho_{ABC},R_{B\to BC}(\rho_{AB}))-2^{-(1/2)I{\left(A:C|B\right)}_{\rho }}, \end{array}$$

where *D*(*A*⊗*B*⊗*C*) denotes the set of states on *A*⊗*B*⊗*C*. We will use the same notation as introduced at the beginning of §4. In addition, we take S to be the set of all states on *A*⊗*B*⊗*C* with a fixed marginal *ρ*_*BC*_ on *B*⊗*C*. The proof proceeds in two steps where we first show that there exists a sequence of recovery maps ${\left\{R_{B\to BC}^{k}\right\}}_{k\in N}$ such that $\lim_{k\to \infty }{\bar{\Delta }}_{R^{k}}(S)\geq 0$, where the property that all elements of S have the same marginal on the *B*⊗*C* system will be important. In the second step, we conclude by an approximation argument that there exists a recovery map $R_{B\to BC}$ such that ${\bar{\Delta }}_{R}(S)\geq 0$.

### (a) Step 1. Existence of a sequence of recovery maps

We start by introducing some notation that is used within this step. Let ${\left\{\Pi_{B}^{b}\right\}}_{b\in N}$ and ${\left\{\Pi_{C}^{c}\right\}}_{c\in N}$ be sequences of finite-rank projectors on *B* and *C* which converge to id_*B*_ and id_*C*_ with respect to the weak operator topology. For any given *ρ*_*ABC*_∈*D*(*A*⊗*B*⊗*C*) consider the normalized projected states

5.2 $$\rho_{ABC}^{b,c}:=\frac{\left({\mathrm{id}}_{A}⊗\Pi_{B}^{b}⊗\Pi_{C}^{c})\rho_{ABC}({\mathrm{id}}_{A}⊗\Pi_{B}^{b}⊗\Pi_{C}^{c}\right)}{\mathrm{tr}(({\mathrm{id}}_{A}⊗\Pi_{B}^{b}⊗\Pi_{C}^{c})\rho_{ABC})}$$

and

5.3 $$\rho_{ABC}^{c}:=\frac{\left({\mathrm{id}}_{A}⊗{\mathrm{id}}_{B}⊗\Pi_{C}^{c})\rho_{ABC}({\mathrm{id}}_{A}⊗{\mathrm{id}}_{B}⊗\Pi_{C}^{c}\right)}{\mathrm{tr}(({\mathrm{id}}_{A}⊗{\mathrm{id}}_{B}⊗\Pi_{C}^{c})\rho_{ABC})},$$

where for any c∈N, the sequence ${\left\{\rho_{ABC}^{b,c}\right\}}_{b\in N}$ converges to $\rho_{ABC}^{c}$ in the trace norm (see, corollary 2 of [43] or lemma 12.1 in the electronic supplementary material) and the sequence ${\left\{\rho_{ABC}^{c}\right\}}_{c\in N}$ converges to *ρ*_*ABC*_ also in the trace norm. Let $S^{b,c}$ be the set of states that is generated by (5.2) for all $\rho_{ABC}\in S$. We note that for any given *b*, *c* all elements of $S^{b,c}$ have an identical marginal on *B*⊗*C*. Let $R_{B\to BC}^{b,c}$ denote a recovery map that satisfies ${\bar{\Delta }}_{R^{b,c}}(S^{b,c})\geq 0$ whose existence is established in the proof of theorem 2.1 for finite-dimensional systems *B* and *C* (see §4). We next state a lemma that explains how ${\bar{\Delta }}_{R}(\rho )$ changes when we replace *ρ* by a projected state *ρ*^*b*,*c*^.

**Lemma 5.1**

*For any ρ*_*BC*_∈*D*(*B*⊗*C*) *there exists a sequence of reals* ${\left\{\xi^{b,c}\right\}}_{b,c\in N}$ *with*^8^ $\lim_{c\to \infty }\lim_{b\to \infty }\xi^{b,c}=0,$ *such that for any* R∈TPCP(B,B⊗C), *any extension ρ*_*ABC*_ *of ρ*_*BC*_, *and* $\rho_{ABC}^{b,c}$ *as given in* (5.2) *we have*

5.4 $${\bar{\Delta }}_{R}(\rho^{b,c})-{\bar{\Delta }}_{R}(\rho )\leq \xi^{b,c}\;\text{for all}\;b,c\in N.$$

**Proof.**

We note that local projections applied to the subsystem *C* can only decrease the mutual information, i.e.

5.5 $$\mathrm{tr}(\Pi_{C}^{c}\rho_{C})I{\left(A:C|B\right)}_{\rho^{c}}\leq I{\left(A:C|B\right)}_{\rho }.$$

To see this assume that a measurement with respect to $\Pi_{C}^{c}$ as well as its orthogonal complement is applied to *ρ*. Furthermore, let *Z* be a random variable that stores the outcome of this measurement. Then by the data processing inequality

5.6 $$\begin{array}{rl} I{\left(A:C|B\right)}_{\rho } & =H{\left(A|B\right)}_{\rho }-H{\left(A|CB\right)}_{\rho }\geq H{\left(A|B\right)}_{\rho^{\prime }}-H{\left(A|CBZ\right)}_{\rho^{\prime }}\\ & \geq H{\left(A|BZ\right)}_{\rho^{\prime }}-H{\left(A|CBZ\right)}_{\rho^{\prime }}=I{\left(A:C|BZ\right)}_{\rho^{\prime }}, \end{array}$$

where *ρ*′ is the state after the measurement. Because *I*(*A*:*C*|*BZ*)_*ρ*′_ can be written as the expectation over the mutual information of the post-measurement states conditioned on the different values of *Z*, and because all these terms are non-negative, the above claim follows.

The Alicki–Fannes inequality [35] ensures that for a fixed finite-dimensional system *C* the conditional mutual information *I*(*A*:*C*|*B*)_*ρ*_=*H*(*C*|*B*)_*ρ*_−*H*(*C*|*AB*)_*ρ*_ is continuous in *ρ* with respect to the trace norm, i.e.

5.7 $$I{\left(A:C|B\right)}_{\rho^{b,c}}\leq I{\left(A:C|B\right)}_{\rho^{c}}+8\varepsilon^{b,c}\log\left(\mathrm{rank}\;\Pi_{C}^{c}\right)+4h(\varepsilon^{b,c}),$$

where $\varepsilon^{b,c}=∥\rho_{ABC}^{b,c}-\rho_{ABC}^{c}{∥}_{1}$ and *h*(⋅) denotes the binary Shannon entropy function defined by $h(p):=-p\log_{2}(p)-(1-p)\log_{2}(1-p)$ for 0≤*p*≤1. Using the Fuchs–van de Graaf inequality [44] and a variant of the gentle-measurement lemma (see lemma 12.1 given in the electronic supplementary material), we find

5.8 $$\varepsilon^{b,c}\leq 2\sqrt{1-F{\left(\rho_{ABC}^{b,c},\rho_{ABC}^{c}\right)}^{2}}\leq 2\sqrt{1-\frac{\mathrm{tr}\left(\Pi_{B}^{b}⊗\Pi_{C}^{c}\rho_{BC}\right)}{\mathrm{tr}(\Pi_{C}^{c}\rho_{C})}}.$$

Combining (5.5) and (5.7) yields

5.9 $$I{\left(A:C|B\right)}_{\rho^{b,c}}\leq \frac{1}{\mathrm{tr}(\Pi_{C}^{c}\rho_{C})}I{\left(A:C|B\right)}_{\rho }+8\varepsilon^{b,c}\log\left(\mathrm{rank}\;\Pi_{C}^{c}\right)+4h(\varepsilon^{b,c}).$$

Since *x*^*y*^≤*x*−*y*+1 for *x*,*y*∈[0,1],^9^ we find

5.10 $$\begin{array}{rl} & 2^{-(1/2)I{\left(A:C|B\right)}_{\rho }}-2^{-(1/2)I{\left(A:C|B\right)}_{\rho^{b,c}}}\\ & \;\leq 2^{-(1/2)I{\left(A:C|B\right)}_{\rho }}-2^{-(1/2)\mathrm{tr}\left(\Pi_{C}^{c}\rho_{C}\right)I{\left(A:C|B\right)}_{\rho^{b,c}}}-\mathrm{tr}\left(\Pi_{C}^{c}\rho_{C}\right)+1. \end{array}$$

According to (5.9) and since $2^{-x}\geq 1-\ln(2)x$ for x∈R, we have

5.11 $$\begin{array}{rl} 2^{-(1/2)\mathrm{tr}\left(\Pi_{C}^{c}\rho_{C}\right)I{\left(A:C|B\right)}_{\rho^{b,c}}} & \geq 2^{-(1/2)I{\left(A:C|B\right)}_{\rho }}2^{-(1/2)\;\mathrm{tr}\left(\Pi_{C}^{c}\rho_{C}\right)\left(8\varepsilon^{b,c}\log\left(\mathrm{rank}\;\Pi_{C}^{c}\right)+4h(\varepsilon^{b,c})\right)}\\ & \geq 2^{-(1/2)I{\left(A:C|B\right)}_{\rho }}-\frac{\ln(2)}{2}\mathrm{tr}\left(\Pi_{C}^{c}\rho_{C}\right)\left(8\varepsilon^{b,c}\log\left(\mathrm{rank}\;\Pi_{C}^{c}\right)+4h(\varepsilon^{b,c})\right). \end{array}$$

Combining (5.10) and (5.11) yields

5.12 $$\begin{array}{rl} & 2^{-(1/2)I{\left(A:C|B\right)}_{\rho }}-2^{-(1/2)I{\left(A:C|B\right)}_{\rho^{b,c}}}\\ & \;\leq \frac{\ln(2)}{2}\mathrm{tr}\left(\Pi_{C}^{c}\rho_{C}\right)\left(8\varepsilon^{b,c}\log\left(\mathrm{rank}\;\Pi_{C}^{c}\right)+4h(\varepsilon^{b,c})\right)+\left(1-\mathrm{tr}\left(\Pi_{C}^{c}\rho_{C}\right)\right)\\ & \;\leq \frac{\ln(2)}{2}\left(8\varepsilon^{b,c}\log\left(\mathrm{rank}\;\Pi_{C}^{c}\right)+4h(\varepsilon^{b,c})\right)+\left(1-\mathrm{tr}\left(\Pi_{C}^{c}\rho_{C}\right)\right). \end{array}$$

For two states *σ*_1_ and *σ*_2_ let $P(\sigma_{1},\sigma_{2}):=\sqrt{1-F{\left(\sigma_{1},\sigma_{2}\right)}^{2}}$ denote the purified distance. Applying the Fuchs–van de Graaf inequality [44] and a variant of the gentle-measurement lemma (see lemma 12.1 in the electronic supplementary material) gives

5.13 $$P{\left(\rho_{ABC},\rho_{ABC}^{b,c}\right)}^{2}=1-F{\left(\rho_{ABC},\rho_{ABC}^{b,c}\right)}^{2}\leq 1-\mathrm{tr}\left(\Pi_{B}^{b}⊗\Pi_{C}^{c}\rho_{BC}\right).$$

Since the purified distance is a metric [45] that is monotonous under trace-preserving completely positive maps [46], theorem 3.4, (5.13) gives

5.14 $$\begin{array}{rl} P\left(\rho_{ABC},R_{B\to BC}(\rho_{AB})\right) & \leq P\left(\rho_{ABC},\rho_{ABC}^{b,c}\right)+P\left(\rho_{ABC}^{b,c},R_{B\to BC}\left(\rho_{AB}^{b,c}\right)\right)\\ & \;+P\left(R_{B\to BC}\left(\rho_{AB}^{b,c}\right),R_{B\to BC}(\rho_{AB})\right)\\ & \leq 2P\left(\rho_{ABC},\rho_{ABC}^{b,c}\right)+P\left(\rho_{ABC}^{b,c},R_{B\to BC}\left(\rho_{AB}^{b,c}\right)\right)\\ & \leq P\left(\rho_{ABC}^{b,c},R_{B\to BC}\left(\rho_{AB}^{b,c}\right)\right)+2\sqrt{1-\mathrm{tr}\left(\Pi_{B}^{b}⊗\Pi_{C}^{c}\rho_{BC}\right)}. \end{array}$$

As the fidelity for states lies between zero and one, (5.14) implies

5.15 $$\begin{array}{rl} & F{\left(\rho_{ABC}^{b,c},R_{B\to BC}\left(\rho_{AB}^{b,c}\right)\right)}^{2}\\ & \;\leq F{\left(\rho_{ABC},R_{B\to BC}(\rho_{AB})\right)}^{2}+4\left(1-\mathrm{tr}\left(\Pi_{B}^{b}⊗\Pi_{C}^{c}\rho_{BC}\right)\right)+4\sqrt{1-\mathrm{tr}\left(\Pi_{B}^{b}⊗\Pi_{C}^{c}\rho_{BC}\right)}\\ & \;\leq F{\left(\rho_{ABC},R_{B\to BC}(\rho_{AB})\right)}^{2}+8\sqrt{1-\mathrm{tr}\left(\Pi_{B}^{b}⊗\Pi_{C}^{c}\rho_{BC}\right)}\\ & \;\leq {\left(F\left(\rho_{ABC},R_{B\to BC}(\rho_{AB})\right)+2\sqrt{2}{\left(1-\mathrm{tr}\left(\Pi_{B}^{b}⊗\Pi_{C}^{c}\rho_{BC}\right)\right)}^{1/4}\right)}^{2}. \end{array}$$

This implies that

5.16 $$F(\rho_{ABC}^{b,c},R_{B\to BC}(\rho_{AB}^{b,c}))\leq F(\rho_{ABC},R_{B\to BC}(\rho_{AB}))+2\sqrt{2}{\left(1-\mathrm{tr}(\Pi_{B}^{b}⊗\Pi_{C}^{c}\rho_{BC})\right)}^{1/4}.$$

By definition of the quantity ${\bar{\Delta }}_{R}(\cdot )$ (see equation (5.1)) the combination of (5.12) and (5.16) yields

5.17 $$\begin{array}{rl} & {\bar{\Delta }}_{R}(\rho^{b,c})-{\bar{\Delta }}_{R}(\rho )\\ & \;\leq \frac{\ln(2)}{2}(8\varepsilon^{b,c}\log(\mathrm{rank}\;\Pi_{C}^{c})+4h(\varepsilon^{b,c}))+(1-\mathrm{tr}(\Pi_{C}^{c}\rho_{C}))+2\sqrt{2}{\left(1-\mathrm{tr}(\Pi_{B}^{b}⊗\Pi_{C}^{c}\rho_{BC})\right)}^{1/4}\\ & \;=:\xi^{b,c}, \end{array}$$

where *ε*^*b*,*c*^ is bounded by (5.8). By a variant of the gentle-measurement lemma (see lemma 12.2 in the electronic supplementary material), we find $\lim_{b\to \infty }\mathrm{tr}(\Pi_{B}^{b}⊗\Pi_{C}^{c}\rho_{BC})=\mathrm{tr}(\Pi_{C}^{c}\rho_{C})$ for all c∈N and hence $\lim_{b\to \infty }\varepsilon^{b,c}=0$ for any c∈N. Furthermore, we have $\lim_{c\to \infty }\mathrm{tr}(\Pi_{C}^{c}\rho_{C})=1$ and $\lim_{c\to \infty }\lim_{b\to \infty }\mathrm{tr}(\Pi_{B}^{b}⊗\Pi_{C}^{c}\rho_{BC})=1$ which implies that $\lim_{c\to \infty }\lim_{b\to \infty }\xi^{b,c}=0$. This proves the assertion. ▪

By lemma 5.1, using the notation defined at the beginning of Step 1, we find

5.18 $$\begin{array}{rl} \underset{c\to \infty }{lim sup}\underset{b\to \infty }{lim sup}{\bar{\Delta }}_{R^{b,c}}(S) & =\underset{c\to \infty }{lim sup}\underset{b\to \infty }{lim sup}\underset{\rho \in S}{\inf}{\bar{\Delta }}_{R^{b,c}}(\rho )\\ & \geq \underset{c\to \infty }{lim sup}\underset{b\to \infty }{lim sup}\underset{\rho \in S}{\inf}\{{\bar{\Delta }}_{R^{b,c}}(\rho^{b,c})-\xi^{b,c}\}\\ & =\underset{c\to \infty }{lim sup}\underset{b\to \infty }{lim sup}\left\{\underset{\rho^{b,c}\in S^{b,c}}{\inf}{\bar{\Delta }}_{R^{b,c}}(\rho^{b,c})\right\}-\xi^{b,c}\\ & =\underset{c\to \infty }{lim sup}\underset{b\to \infty }{lim sup}{\bar{\Delta }}_{R^{b,c}}(S^{b,c})\\ & \geq 0, \end{array}$$

where the second equality step is valid since all states in S have the same fixed marginal on *B*⊗*C* and since the sequence ${\left\{\xi^{b,c}\right\}}_{b,c\in N}$ only depends on this marginal. The penultimate step uses that $\lim_{c\to \infty }\lim_{b\to \infty }\xi^{b,c}=0$. The final inequality follows by definition of $R_{B\to BC}^{b,c}$. Inequality (5.18) implies that there exist sequences ${\left\{b_{k}\right\}}_{k\in N}$ and ${\left\{c_{k}\right\}}_{k\in N}$ such that $\underset{k\to \infty }{lim sup}\Delta_{R^{b_{k},c_{k}}}(S)\geq 0$. Setting $R_{B\to BC}^{k}=R_{B\to BC}^{b_{k},c_{k}}$ then implies that there exists a sequence ${\left\{R_{B\to BC}^{k}\right\}}_{k\in N}$ of recovery maps that satisfies

5.19 $$\underset{k\to \infty }{lim sup}{\bar{\Delta }}_{R^{k}}(S)\geq 0.$$

### (b) Step 2. Existence of a limit

Recall that S is the set of density operators on *A*⊗*B*⊗*C* with a fixed marginal *ρ*_*BC*_ on *B*⊗*C*. The goal of this step is to use (5.19) to prove that there exists a recovery map $R_{B\to BC}$ such that

5.20 $${\bar{\Delta }}_{R}(S)\geq 0.$$

Let ${\left\{\Pi_{B}^{m}\right\}}_{m\in N}$ and ${\left\{\Pi_{C}^{m}\right\}}_{m\in N}$ be sequences of projectors with rank *m* that weakly converge to id_*B*_ and id_*C*_, respectively. Furthermore, for any *m* and any R∈TPCP(B,B⊗C) let ${\left[R\right]}^{m}$ be the trace non-increasing map obtained from R by projecting the input and output with $\Pi_{B}^{m}$ and $\Pi_{B}^{m}⊗\Pi_{C}^{m}$, respectively. We start with a preparatory lemma that proves a relationship between ${\bar{\Delta }}_{{\left[R\right]}^{m}}(S)$ and ${\bar{\Delta }}_{R}(S)$.

**Lemma 5.2**

*For any ρ*_*BC*_∈*D*(*B*⊗*C*) *there exists a sequence of reals* ${\left\{\delta^{m}\right\}}_{m\in N}$ *with* $\lim_{m\to \infty }\delta^{m}=0$,^10^ *such that for any* R∈TPCP(B,B⊗C) *we have*

5.21 $${\bar{\Delta }}_{{\left[R\right]}^{m}}(S)\geq {\bar{\Delta }}_{R}(S)-\delta^{m}-4\varepsilon^{1/4},$$

*where* $∥R(\rho_{B})-\rho_{BC}{∥}_{1}\leq \varepsilon$.

**Proof.**

For any $\rho_{ABC}\in S$ and any m∈N let us define the non-negative operator ${\hat{\rho }}_{AB}^{m}:=({\mathrm{id}}_{A}⊗\Pi_{B}^{m})\rho_{AB}$ $\left({\mathrm{id}}_{A}⊗\Pi_{B}^{m}\right)$. By definition of ${\bar{\Delta }}_{R}(\cdot )$ (see equation (5.1)), it suffices to show that for any $\rho_{ABC}\in S$, any R∈TPCP(B,B⊗C), *ε*∈[0,2] such that $∥R(\rho_{B})-\rho_{BC}{∥}_{1}\leq \varepsilon$ and

5.22 $${\tilde{\rho }}_{ABC}^{m}:=({\mathrm{id}}_{A}⊗\Pi_{B}^{m}⊗\Pi_{C}^{m})R_{B\to BC}({\hat{\rho }}_{AB}^{m})({\mathrm{id}}_{A}⊗\Pi_{B}^{m}⊗\Pi_{C}^{m})$$

we have $F(\rho_{ABC},{\tilde{\rho }}_{ABC}^{m})\geq F(\rho_{ABC},R_{B\to BC}(\rho_{AB}))-\delta^{m}-4\varepsilon^{1/4}$. As in Step 1, let *P*(⋅,⋅) denote the purified distance. A variant of the gentle-measurement lemma (see lemma 12.1 in the electronic supplementary material) implies that

5.23 $$P{\left(\rho_{AB},{\hat{\rho }}_{AB}^{m}\right)}^{2}=1-F{\left(\rho_{AB},{\hat{\rho }}_{AB}^{m}\right)}^{2}\leq 1-\mathrm{tr}{\left(\rho_{B}\Pi_{B}^{m}\right)}^{2}.$$

Similarly, we obtain

5.24 $$\begin{array}{rl} P{\left(R_{B\to BC}({\hat{\rho }}_{AB}^{m}),{\tilde{\rho }}_{ABC}^{m}\right)}^{2} & \leq 1-\mathrm{tr}{\left(R_{B\to BC}({\hat{\rho }}_{AB}^{m})\Pi_{B}^{m}⊗\Pi_{C}^{m}\right)}^{2}\\ & =1-\mathrm{tr}{\left(R_{B\to BC}({\hat{\rho }}_{B}^{m})\Pi_{B}^{m}⊗\Pi_{C}^{m}\right)}^{2}. \end{array}$$

By Hölder’s inequality, monotonicity of the trace norm for trace-preserving completely positive maps [47], example 9.1.8 and corollary 9.1.10 and (5.23) together with the Fuchs–van de Graaf inequality [44] and a variant of the gentle-measurement lemma (see lemma 12.1 given in the electronic supplementary material), we find

5.25 $$\begin{array}{rl} |\mathrm{tr}((R_{B\to BC}({\hat{\rho }}_{B}^{m})-R_{B\to BC}(\rho_{B}))\Pi_{B}^{m}⊗\Pi_{C}^{m})| & \leq ∥R_{B\to BC}(\rho_{B})-R_{B\to BC}({\hat{\rho }}_{B}^{m}){∥}_{1}∥\Pi_{B}^{m}⊗\Pi_{C}^{m}{∥}_{\infty }\\ & =∥R_{B\to BC}(\rho_{B})-R_{B\to BC}({\hat{\rho }}_{B}^{m}){∥}_{1}\leq ∥\rho_{B}-{\hat{\rho }}_{B}^{m}{∥}_{1}\\ & \leq ∥\rho_{AB}-{\hat{\rho }}_{AB}^{m}{∥}_{1}\leq 2\sqrt{1-\mathrm{tr}{\left(\rho_{B}\Pi_{B}^{m}\right)}^{2}}. \end{array}$$

Combining (5.24), (5.25) and Hölder’s inequality together with the assumption $∥R(\rho_{B})-\rho_{BC}{∥}_{1}\leq \varepsilon$ gives

5.26 $$\begin{array}{rl} P{\left(R_{B\to BC}({\hat{\rho }}_{AB}^{m}),{\tilde{\rho }}_{ABC}^{m}\right)}^{2} & \leq 1-\mathrm{tr}{\left(R_{B\to BC}(\rho_{B})\Pi_{B}^{m}⊗\Pi_{C}^{m}\right)}^{2}+4\sqrt{1-\mathrm{tr}{\left(\rho_{B}\Pi_{B}^{m}\right)}^{2}}\\ & \leq 1-\mathrm{tr}{\left(\rho_{BC}\Pi_{B}^{m}⊗\Pi_{C}^{m}\right)}^{2}+4\sqrt{1-\mathrm{tr}{\left(\rho_{B}\Pi_{B}^{m}\right)}^{2}}+2\varepsilon . \end{array}$$

Inequalities (5.23), (5.26) and the monotonicity of the purified distance under trace-preserving and completely positive maps [46], Theorem 3.4 show that

5.27 $$\begin{array}{rl} P(\rho_{ABC},{\tilde{\rho }}_{ABC}^{m}) & \leq P(\rho_{ABC},R_{B\to BC}(\rho_{AB}))+P(R_{B\to BC}(\rho_{AB}),R_{B\to BC}({\hat{\rho }}_{AB}^{m}))+P(R_{B\to BC}({\hat{\rho }}_{AB}^{m}),{\tilde{\rho }}_{ABC}^{m})\\ & \leq P(\rho_{ABC},R_{B\to BC}(\rho_{AB}))+P(\rho_{AB},{\hat{\rho }}_{AB}^{m})+P(R_{B\to BC}({\hat{\rho }}_{AB}^{m}),{\tilde{\rho }}_{ABC}^{m})\\ & \leq P(\rho_{ABC},R_{B\to BC}(\rho_{AB}))+\frac{{\left(\delta^{m}\right)}^{2}}{8}+\sqrt{2\varepsilon }, \end{array}$$

for

5.28 $$\delta^{m}:=\sqrt{8}{\left(\sqrt{1-\mathrm{tr}{\left(\rho_{B}\Pi_{B}^{m}\right)}^{2}}+\sqrt{1-\mathrm{tr}{\left(\rho_{BC}\Pi_{B}^{m}⊗\Pi_{C}^{m}\right)}^{2}+4\sqrt{1-\mathrm{tr}{\left(\rho_{B}\Pi_{B}^{m}\right)}^{2}}}\right)}^{1/2}.$$

As the purified distance between two states lies inside the interval [0,1] and since ${\left(\delta^{m}\right)}^{2}/8+\sqrt{2\varepsilon }\in [0,6]$, (5.27) implies that whenever $F{\left(\rho_{ABC},R_{B\to BC}(\rho_{AB})\right)}^{2}\geq {\left(\delta^{m}\right)}^{2}+8\sqrt{2\varepsilon }$, we have

5.29 $$\begin{array}{rl} F{\left(\rho_{ABC},{\tilde{\rho }}_{ABC}^{m}\right)}^{2} & \geq F{\left(\rho_{ABC},R_{B\to BC}(\rho_{AB})\right)}^{2}-{\left(\delta^{m}\right)}^{2}-8\sqrt{2\varepsilon }\\ & \geq {\left(F(\rho_{ABC},R_{B\to BC}(\rho_{AB}))-\sqrt{{\left(\delta^{m}\right)}^{2}+8\sqrt{2\varepsilon }}\right)}^{2}. \end{array}$$

As a result, we find

5.30 $$F(\rho_{ABC},{\tilde{\rho }}_{ABC}^{m})\geq F(\rho_{ABC},R_{B\to BC}(\rho_{AB}))-\delta^{m}-\sqrt{8}{\left(2\varepsilon \right)}^{1/4},$$

which proves (5.21) since $\sqrt{8}2^{1/4}\leq 4$.

Recall that *B* and *C* are separable Hilbert spaces and that ${\left\{\Pi_{B}^{m}\right\}}_{m\in N}$ and ${\left\{\Pi_{B}^{m}⊗\Pi_{C}^{m}\right\}}_{m\in N}$ converge weakly to id_*B*_ and id_*B*_⊗id_*C*_, respectively. A variant of the gentle-measurement lemma (see lemma 12.2 given in the electronic supplementary material) thus shows that $\lim_{m\to \infty }\delta^{m}=0$ since $\lim_{m\to \infty }\;\mathrm{tr}(\rho_{B}\Pi_{B}^{m})=1$ and $\lim_{m\to \infty }\mathrm{tr}(\rho_{BC}\Pi_{B}^{m}⊗\Pi_{C}^{m})=1$. ▪

The following lemma proves that for sufficiently large *m* and a recovery map $R_{B\to BC}$ that maps *ρ*_*B*_ to density operators that are close to *ρ*_*BC*_, the operator ${\left[R\right]}^{m}(\rho_{AB})$ has a trace that is bounded from below by essentially one.

**Lemma 5.3**

*Let A*,*B and C be separable Hilbert spaces. For any density operator ρ*_*AB*_∈*D*(*A*⊗*B*) *and any* R∈TPCP(B,B⊗C), *we have*

5.31 $$\mathrm{tr}({\left[R\right]}^{m}(\rho_{AB}))\geq \mathrm{tr}(\Pi_{B}^{m}⊗\Pi_{C}^{m}\rho_{BC})-2\sqrt{1-\mathrm{tr}(\Pi_{B}^{m}\rho_{B})}-∥R(\rho_{B})-\rho_{BC}{∥}_{1}.$$

**Proof.**

We first note that by Hölder’s inequality and monotonicity of the trace norm for trace-preserving completely positive maps [47], example 9.1.8 and corollary 9.1.10 we have

5.32 $$\begin{array}{rl} & |\mathrm{tr}(\Pi_{B}^{m}⊗\Pi_{C}^{m}(R(\rho_{B})-R(\Pi_{B}^{m}\rho_{B}\Pi_{B}^{m})))|\\ & \;\leq ∥R(\rho_{B})-R(\Pi_{B}^{m}\rho_{B}\Pi_{B}^{m}){∥}_{1}\leq ∥\rho_{B}-\Pi_{B}^{m}\rho_{B}\Pi_{B}^{m}{∥}_{1}. \end{array}$$

Together with Hölder’s inequality this implies

5.33 $$\begin{array}{rl} \mathrm{tr}({\left[R\right]}^{m}(\rho_{AB})) & =\mathrm{tr}(\Pi_{B}^{m}⊗\Pi_{C}^{m}R(\Pi_{B}^{m}\rho_{AB}\Pi_{B}^{m}))=\mathrm{tr}(\Pi_{B}^{m}⊗\Pi_{C}^{m}R(\Pi_{B}^{m}\rho_{B}\Pi_{B}^{m}))\\ & \geq \mathrm{tr}(\Pi_{B}^{m}⊗\Pi_{C}^{m}R(\rho_{B}))-∥\rho_{B}-\Pi_{B}^{m}\rho_{B}\Pi_{B}^{m}{∥}_{1}\\ & \geq \mathrm{tr}(\Pi_{B}^{m}⊗\Pi_{C}^{m}\rho_{BC})-∥\rho_{B}-\Pi_{B}^{m}\rho_{B}\Pi_{B}^{m}{∥}_{1}-∥R(\rho_{B})-\rho_{BC}{∥}_{1}. \end{array}$$

Combining a generalization of the Fuchs–van de Graaf inequality (see lemma 8.2 in the electronic supplementary material) and a variant of the gentle-measurement lemma (see lemma 12.1 in the electronic supplementary material) gives

5.34 $$\begin{array}{rl} ∥\rho_{B}-\Pi_{B}^{m}\rho_{B}\Pi_{B}^{m}{∥}_{1} & \leq 2\sqrt{1-F{\left(\rho_{B},\Pi_{B}^{m}\rho_{B}\Pi_{B}^{m}\right)}^{2}}=2\sqrt{1-\mathrm{tr}(\Pi_{B}^{m}\rho_{B})F{\left(\frac{\rho_{B},\Pi_{B}^{m}\rho_{B}\Pi_{B}^{m}}{\mathrm{tr}(\Pi_{B}^{m}\rho_{B})}\right)}^{2}}\\ & \leq 2\sqrt{1-\mathrm{tr}(\Pi_{B}^{m}\rho_{B})}, \end{array}$$

which together with (5.33) proves the assertion. ▪

According to (5.19) the mappings $R^{k}$ satisfy

5.35 $${\bar{\Delta }}_{R^{k}}(S)\geq -{\tilde{\varepsilon }}^{k},$$

with ${\tilde{\varepsilon }}^{k}\geq 0$ such that $\underset{k\to \infty }{lim inf}{\tilde{\varepsilon }}^{k}=0$. As explained in remark 2.3, by considering a state ${\bar{\rho }}_{ABC}=\rho_{A}⊗\rho_{BC}\in S$, (5.35) implies $F(\rho_{BC},R^{k}(\rho_{B}))\geq -{\tilde{\varepsilon }}^{k}+1$. Applying the Fuchs–van de Graaf inequality [44] gives

5.36 $$∥\rho_{BC}-R^{k}(\rho_{B}){∥}_{1}\leq 2\sqrt{{\tilde{\varepsilon }}^{k}(2-{\tilde{\varepsilon }}^{k})}=:\varepsilon^{k},$$

where $\underset{k\to \infty }{lim inf}\varepsilon^{k}=0$ because $\underset{k\to \infty }{lim inf}{\tilde{\varepsilon }}^{k}=0$.

By lemma 5.2, we have

5.37 $${\bar{\Delta }}_{{\left[R^{k}\right]}^{m}}(S)\geq {\bar{\Delta }}_{R^{k}}(S)-4{\left(\varepsilon^{k}\right)}^{1/4}-\delta^{m}.$$

Hence, using our starting point (5.19),

5.38 $$\underset{k\to \infty }{lim sup}{\bar{\Delta }}_{{\left[R^{k}\right]}^{m}}(S)\geq \underset{k\to \infty }{lim sup}{\bar{\Delta }}_{R^{k}}(S)-4{\left(\varepsilon^{k}\right)}^{1/4}-\delta^{m}\geq -\delta^{m}.$$

Because, for any fixed m∈N, the mappings ${\left[R^{k}\right]}^{m}$, for k∈N, are all contained in the same finite-dimensional subspace (i.e. the set of trace non-increasing maps from operators on the support of $\Pi_{B}^{m}$ to operators on the support of $\Pi_{B}^{m}⊗\Pi_{C}^{m}$), and because the space of all such mappings is compact (see remark 10.3 in the electronic supplementary material), for any fixed m∈N there exists a subsequence of the sequence ${\left\{{\left[R^{k}\right]}^{m}\right\}}_{k\in N}$ that converges. Specifically for any fixed m∈N there exists a sequence ${\left\{k_{i}^{m}\right\}}_{i\in N}$ such that

5.39 $${\bar{R}}^{m}:=\lim_{i\to \infty }{\left[R^{k_{i}^{m}}\right]}^{m}$$

is well defined. Furthermore, because of the continuity of $R\mapsto {\bar{\Delta }}_{R}(\rho_{ABC})$ on the set of maps from operators on the support of $\Pi_{B}^{m}$ to operators on the support of $\Pi_{B}^{m}⊗\Pi_{C}^{m}$ (see lemma 10.4 given in the electronic supplementary material), we have

5.40 $$\begin{array}{rl} {\bar{\Delta }}_{{\bar{R}}^{m}}(S) & =\underset{\rho \in S}{\inf}{\bar{\Delta }}_{{\bar{R}}^{m}}(\rho )=\underset{\rho \in S}{\inf}\lim_{i\to \infty }{\bar{\Delta }}_{{\left[R^{k_{i}^{m}}\right]}^{m}}(\rho )\geq \underset{i\to \infty }{lim sup}\underset{\rho \in S}{\inf}{\bar{\Delta }}_{{\left[R^{k_{i}^{m}}\right]}^{m}}(\rho )\\ & =\underset{i\to \infty }{lim sup}{\bar{\Delta }}_{{\left[R^{k_{i}^{m}}\right]}^{m}}(S)\geq -\delta^{m}, \end{array}$$

and, hence,

5.41 $$\underset{m\to \infty }{lim inf}{\bar{\Delta }}_{{\bar{R}}^{m}}(S)\geq 0.$$

Without loss of generality, we can assume that the projector $\Pi_{B}^{m}$ is in the eigenbasis of *ρ*_*B*_ that is denoted by $\left\{{|b\rangle }_{B}^{m}\right\}$. For a basis $\left\{{|b\rangle }_{\bar{B}}^{m}\right\}$, we define the projector $\Pi_{\bar{B}}^{m}=W\Pi_{B}^{m}W^{†}$ for an isometry $W=\sum_{b}|b\rangle \langle b{|}_{\bar{B}}^{m}$. For any m∈N, let $\rho_{BC:\bar{B}}^{m}$ be the operator obtained by applying ${\bar{R}}^{m}$ to a purification $\rho_{B:\bar{B}}=(\rho_{B}^{1/2}⊗{\mathrm{id}}_{\bar{B}})\sum_{b}{|b\rangle }_{B}⊗{|b\rangle }_{\bar{B}}$ of *ρ*_*B*_.

As explained above, there exists a converging subsequence ${\left\{k_{i}^{m+1}\right\}}_{i\in N}$ of ${\left\{k_{i}^{m+1}\right\}}_{i\in N}$ such that ${\bar{R}}^{m}:=\lim_{i\to \infty }{\left[R^{k_{i}^{m+1}}\right]}^{m}$. Using the definition of ${\bar{R}}^{m}$ and that $\Pi_{B}^{m}\leq \Pi_{B}^{m^{\prime }}$, $\Pi_{C}^{m}\leq \Pi_{C}^{m^{\prime }}$ and $\Pi_{\bar{B}}^{m}\leq \Pi_{\bar{B}}^{m^{\prime }}$ for *m*≤*m*′, we obtain

5.42 $$\begin{array}{rl} \rho_{BC:\bar{B}}^{m} & ={\bar{R}}^{m}(\rho_{B:\bar{B}})\\ & =\lim_{i\to \infty }{\left[R^{k_{i}^{m+1}}\right]}^{m}(\rho_{B:\bar{B}})=\lim_{i\to \infty }(\Pi_{B}^{m}⊗\Pi_{C}^{m}){\left[R^{k_{i}^{m+1}}\right]}^{m+1}(\Pi_{B}^{m}\rho_{B:\bar{B}}\Pi_{B}^{m})(\Pi_{B}^{m}⊗\Pi_{C}^{m})\\ & =\lim_{i\to \infty }(\Pi_{B}^{m}⊗\Pi_{C}^{m}⊗\Pi_{\bar{B}}^{m}){\left[R^{k_{i}^{m+1}}\right]}^{m+1}(\rho_{B:\bar{B}})(\Pi_{B}^{m}⊗\Pi_{C}^{m}⊗\Pi_{\bar{B}}^{m})\\ & =(\Pi_{B}^{m}⊗\Pi_{C}^{m}⊗\Pi_{\bar{B}}^{m}){\bar{R}}^{m+1}(\rho_{B:\bar{B}})(\Pi_{B}^{m}⊗\Pi_{C}^{m}⊗\Pi_{\bar{B}}^{m})\\ & =(\Pi_{B}^{m}⊗\Pi_{C}^{m}⊗\Pi_{\bar{B}}^{m})\rho_{BC:\bar{B}}^{m+1}(\Pi_{B}^{m}⊗\Pi_{C}^{m}⊗\Pi_{\bar{B}}^{m}). \end{array}$$

As a result, since $\Pi_{B}^{m}\leq \Pi_{B}^{m^{\prime }}$, $\Pi_{C}^{m}\leq \Pi_{C}^{m^{\prime }}$ and $\Pi_{\bar{B}}^{m}\leq \Pi_{\bar{B}}^{m^{\prime }}$ for *m*≤*m*′, we have for any *m*≤*m*′

5.43 $$\rho_{BC:\bar{B}}^{m}=(\Pi_{B}^{m}⊗\Pi_{C}^{m}⊗\Pi_{\bar{B}}^{m})\rho_{BC:\bar{B}}^{m^{\prime }}(\Pi_{B}^{m}⊗\Pi_{C}^{m}⊗\Pi_{\bar{B}}^{m}).$$

A variant of the gentle-measurement lemma (see lemma 12.1 in the electronic supplementary material) together with (5.43) implies

5.44 $$\begin{array}{rl} F(\rho_{BC:\bar{B}}^{m},\rho_{BC:\bar{B}}^{m^{\prime }}) & =F(\Pi_{B}^{m}⊗\Pi_{C}^{m}⊗\Pi_{\bar{B}}^{m}\rho_{BC:\bar{B}}^{m^{\prime }}\Pi_{B}^{m}⊗\Pi_{C}^{m}⊗\Pi_{\bar{B}}^{m},\rho_{BC:\bar{B}}^{m^{\prime }})\\ & \geq \mathrm{tr}(\rho_{BC:\bar{B}}^{m^{\prime }}\Pi_{B}^{m}⊗\Pi_{C}^{m}⊗\Pi_{\bar{B}}^{m})=\mathrm{tr}(\rho_{BC:\bar{B}}^{m}). \end{array}$$

A generalization of the Fuchs–van de Graaf inequality (see lemma 8.2 in the electronic supplementary material) yields for *m*′≥*m*

5.45 $$∥\rho_{BC:\bar{B}}^{m}-\rho_{BC:\bar{B}}^{m^{\prime }}{∥}_{1}\leq 2\sqrt{\mathrm{tr}{\left(\rho_{BC:\bar{B}}^{m^{\prime }}\right)}^{2}-F{\left(\rho_{BC:\bar{B}}^{m},\rho_{BC:\bar{B}}^{m^{\prime }}\right)}^{2}}\leq 2\sqrt{\mathrm{tr}{\left(\rho_{BC:\bar{B}}^{m^{\prime }}\right)}^{2}-\mathrm{tr}{\left(\rho_{BC:\bar{B}}^{m}\right)}^{2}}.$$

We now prove that as m→∞, $\mathrm{tr}(\rho_{BC:\bar{B}}^{m})$ goes to 1. Note that since *B* is a separable Hilbert space and $\rho_{B:\bar{B}}$ is normalized it can be written as $\rho_{B:\bar{B}}=|\psi \rangle \langle \psi |$, where |*ψ*〉 is a state on $B⊗\bar{B}$. Furthermore, as $\Pi_{B}^{m}⊗\Pi_{C}^{m}⊗\Pi_{\bar{B}}^{m}\leq {\mathrm{id}}_{BC\bar{B}}$, (5.43) implies that

5.46 $$\mathrm{tr}(\rho_{BC:\bar{B}}^{m})\leq \mathrm{tr}(\rho_{BC:\bar{B}}^{m^{\prime }})\leq 1\;\mathrm{for}\;m^{\prime }\geq m.$$

By definition of $\rho_{BC:\bar{B}}^{m}$, lemma 5.3 together with (5.36) implies that

5.47 $$\begin{array}{rl} \lim_{m\to \infty }\mathrm{tr}(\rho_{BC:\bar{B}}^{m}) & =\lim_{m\to \infty }\lim_{i\to \infty }\mathrm{tr}({\left[R^{k_{i}^{m}}\right]}^{m}(\rho_{B:\bar{B}}))\\ & \geq \lim_{m\to \infty }\mathrm{tr}(\Pi_{B}^{m}⊗\Pi_{C}^{m}\rho_{BC})-∥\rho_{B}-\Pi_{B}^{m}\rho_{B}\Pi_{B}^{m}{∥}_{1}-\underset{i\to \infty }{lim inf}\varepsilon^{k_{i}^{m}}\\ & \geq \lim_{m\to \infty }\mathrm{tr}(\Pi_{B}^{m}⊗\Pi_{C}^{m}\rho_{BC})-2\sqrt{1-\mathrm{tr}{\left(\Pi_{B}^{m}\rho_{B}\right)}^{2}}=1, \end{array}$$

where the second inequality uses a generalized version of the Fuchs–van de Graaf inequality (see lemma 8.2 in the electronic supplementary material), a variant of the gentle-measurement lemma (see lemma 12.1 in the electronic supplementary material), and that $\underset{i\to \infty }{lim inf}\varepsilon^{k_{i}^{m}}=0$ for all m∈N. The final step follows by another variant of the gentle-measurement lemma (see lemma 12.2 in the electronic supplementary material).

Equations (5.45)–(5.47) show that, ${\left\{\rho_{BC:\bar{B}}^{m}\right\}}_{m\in N}$ is a Cauchy sequence. Because the set of sub-normalized non-negative operators (i.e. the set of sub-normalized density operators) is complete,^11^ this sequence converges towards such an operator, i.e. we can define a density operator

5.48 $${\tilde{\rho }}_{BC:\bar{B}}:=\lim_{m\to \infty }\rho_{BC:\bar{B}}^{m}.$$

We note that the operators $\rho_{BC:\bar{B}}^{m}$ are not normalized in general. However, (5.47) shows that ${\tilde{\rho }}_{BC:\bar{B}}$ has unit trace. We now define the recovery map $R_{B\to BC}$ as the one that maps $\rho_{B:\bar{B}}$ to ${\tilde{\rho }}_{BC:\bar{B}}$. We note that this does not uniquely define the recovery map $R_{B\to BC}$, which is not a problem as theorem 2.1 proves the *existence* of a recovery map that satisfies (2.1) and does not claim that this map is unique. It remains to show that $R_{B\to BC}$ has the property (5.20). This follows from the observation that any density operator *ρ*_*AB*_ can be obtained from the purification $\rho_{B:\bar{B}}$ by applying a trace-preserving completely positive map $T_{\bar{B}\to A}$ from $\bar{B}$ to *A*. By a continuity property stated in lemma 10.5 in the electronic supplementary material and because $T_{\bar{B}\to A}$ commutes with any recovery map $R_{B\to BC}$ from *B* to *B*⊗*C*, we have

5.49 $$\begin{array}{rl} R_{B\to BC}(\rho_{AB}) & =(R_{B\to BC}\circ T_{\bar{B}\to A})(\rho_{B:\bar{B}})=(T_{\bar{B}\to A}\circ R_{B\to BC})(\rho_{B:\bar{B}})=T_{\bar{B}\to A}({\tilde{\rho }}_{BC:\bar{B}})\\ & =T_{\bar{B}\to A}(\lim_{m\to \infty }\rho_{BC:\bar{B}}^{m})=\lim_{m\to \infty }T_{\bar{B}\to A}(\rho_{BC:\bar{B}}^{m})=\lim_{m\to \infty }(T_{\bar{B}\to A}\circ {\bar{R}}_{B\to BC}^{m})(\rho_{B:\bar{B}})\\ & =\lim_{m\to \infty }({\bar{R}}_{B\to BC}^{m}\circ T_{\bar{B}\to A})(\rho_{B:\bar{B}})=\lim_{m\to \infty }{\bar{R}}_{B\to BC}^{m}(\rho_{AB}). \end{array}$$

Using the continuity of the fidelity [12,13], this implies that

5.50 $${\bar{\Delta }}_{R}(\rho )=\lim_{m\to \infty }{\bar{\Delta }}_{{\bar{R}}^{m}}(\rho ),$$

for any ρ∈S. Combining this with (5.41) gives

5.51 $${\bar{\Delta }}_{R}(S)=\underset{\rho \in S}{\inf}{\bar{\Delta }}_{R}(\rho )=\underset{\rho \in S}{\inf}\lim_{m\to \infty }{\bar{\Delta }}_{{\bar{R}}^{m}}(\rho )\geq \underset{m\to \infty }{lim inf}\underset{\rho \in S}{\inf}{\bar{\Delta }}_{{\bar{R}}^{m}}(\rho )=\underset{m\to \infty }{lim inf}{\bar{\Delta }}_{{\bar{R}}^{m}}(S)\geq 0,$$

which concludes Step 2 and thus completes the proof of theorem 2.1 in the general case where *B* and *C* are no longer finite-dimensional.

## 6. Proof of corollary thm2.4

The first statement of corollary 2.4 that holds for separable Hilbert spaces follows immediately from theorem 2.1, since $2^{-(1/2)I{\left(A:C|B\right)}_{\rho }}\geq 1-(\ln(2)/2)I{\left(A:C|B\right)}_{\rho }$. The proof of the second statement of corollary 2.4 is partitioned into three steps.^12^ We first show that a similar method as used in §4 can be used to reveal certain insights about the structure of the recovery map $R_{B\to BC}$ (which is not universal) that satisfies

6.1 $$F(\rho_{ABC},R_{B\to BC}(\rho_{AB}))\geq 1-\frac{\ln(2)}{2}I{\left(A:C|B\right)}_{\rho }.$$

In a second step, by invoking proposition 4.1, we use this knowledge to prove that for a fixed *A* system there exists a recovery map that satisfies (6.1) which is universal and preserves the structure of the non-universal recovery map from before. Finally, in Step 3 we show how the dependency on the fixed *A* system can be removed.

### (a) Step 1. Structure of a non-universal recovery map

We will show that for any density operator *ρ*_*ABC*_ on *A*⊗*B*⊗*C*, where *A*, *B*, and *C* are finite-dimensional Hilbert spaces there exists a trace-preserving completely positive map $R_{B\to BC}$ that satisfies (6.1) and is of the form

6.2 $$X_{B}\mapsto \rho_{BC}^{1/2}W_{BC}(\rho_{B}^{-1/2}X_{B}\rho_{B}^{-1/2}⊗{\mathrm{id}}_{C})W_{BC}^{†}\rho_{BC}^{1/2},$$

on the support of *ρ*_*B*_, where *W*_*BC*_ is a unitary on *B*⊗*C*. We start by proving the following preparatory lemma.

**Lemma 6.1**

*For any density operator ρ*_*ABC*_ *on A*⊗*B*⊗*C*, *where A*, *B* *and C are finite-dimensional Hilbert spaces there exists a trace-preserving completely positive map* $R_{B\to BC}$ *of the form*

6.3 $$X_{B}\mapsto V_{BC}\rho_{BC}^{1/2}(\rho_{B}^{-1/2}U_{B}X_{B}U_{B}^{†}\rho_{B}^{-1/2}⊗{\mathrm{id}}_{C})\rho_{BC}^{1/2}V_{BC}^{†},$$

*where V*_*BC*_ *is a unitary on B*⊗*C that commutes with* *ρ*_*BC*_ *and U*_*B*_ *is a unitary on B that commutes with ρ*_*B*_ *such that*

6.4 $$F(\rho_{ABC},R_{B\to BC}(\rho_{AB}))\geq 1-\frac{\ln(2)}{2}I{\left(A:C|B\right)}_{\rho }.$$

**Proof.**

Let *ρ*_*ABC*_ be an arbitrary state on *A*⊗*B*⊗*C* and let $\rho_{ABC}^{0}$ be a Markov chain with the same marginal on the *B*⊗*C* system, i.e. $\rho_{BC}^{0}=\rho_{BC}$. For *p*∈(0,1], define the state

6.5 $$\rho_{\hat{A}ABC}^{p}:=(1-p)|0\rangle \langle 0{|}_{\hat{A}}⊗\rho_{ABC}^{0}+p|1\rangle \langle 1{|}_{\hat{A}}⊗\rho_{ABC}.$$

The main result of [6] (see theorem 5.1 and remark 4.3 in [6]) implies that there exists a recovery map $R_{B\to BC}$ of the form

6.6 $$X_{B}\mapsto V_{BC}\rho_{BC}^{1/2}(\rho_{B}^{-1/2}U_{B}X_{B}U_{B}^{†}\rho_{B}^{-1/2}⊗{\mathrm{id}}_{C})\rho_{BC}^{1/2}V_{BC}^{†},$$

where *U*_*B*_ is diagonal with respect to the eigenbasis of *ρ*_*B*_, $U_{B}U_{B}^{†}\leq {\mathrm{id}}_{B}$ and *V*_*BC*_ is a unitary on *B*⊗*C*, such that

6.7 $$F\left(\rho_{\hat{A}ABC}^{p},R_{B\to BC}(\rho_{\hat{A}AB}^{p})\right)\geq 1-\frac{\ln(2)}{2}I{\left(\hat{A}A:C|B\right)}_{\rho^{p}}.$$

(Alternatively this statement also follows from [20]—which however appeared after the completion of this work.) By lemma 6.2, using that *I*(*A*:*C*|*B*)_*ρ*^0^_=0 since $\rho_{ABC}^{0}$ is a Markov chain, this may be rewritten as

6.8 $$\begin{array}{rl} & p(1-F(\rho_{ABC},R_{B\to BC}(\rho_{AB})))+(1-p)(1-F(\rho_{ABC}^{0},R_{B\to BC}(\rho_{AB}^{0})))\\ & \;\leq p\frac{\ln(2)}{2}I{\left(A:C|B\right)}_{\rho }. \end{array}$$

Let us assume by contradiction that any recovery map $R_{B\to BC}$ that satisfies (6.8) does not leave $\rho_{ABC}^{0}$ invariant, i.e. $\rho_{ABC}^{0}\neq R_{B\to BC}(\rho_{AB}^{0})$. This implies that there exists a $\delta_{R}\in (0,1]$, which may depend on the recovery map $R_{B\to BC}$, such that $1-F(\rho_{ABC}^{0},R_{B\to BC}(\rho_{AB}^{0}))=\delta_{R}$. In the following, we argue that there exists a universal (i.e. independent of $R_{B\to BC}$) constant *δ*∈(0,1] such that $1-F(\rho_{ABC}^{0},R_{B\to BC}(\rho_{AB}^{0}))\geq \delta$ for all recovery maps $R_{B\to BC}$ that satisfy (6.8). Since the set of trace-preserving completely positive maps from *B* to *B*⊗*C* that satisfy (6.8) is compact^13^ and the function $f:\mathrm{TPCP}(B,B⊗C)∋R_{B\to BC}\mapsto 1-F(\rho_{ABC}^{0},R_{B\to BC}(\rho_{AB}^{0}))\in [0,1]$ is continuous (see lemma 10.4 in the electronic supplementary material), Weierstrass’ theorem ensures that $\delta :=\min_{R_{B\to BC}}f(R_{B\to BC})$, where we optimize over the set of trace-preserving completely positive maps from *B* to *B*⊗*C* that satisfy (6.8), exists. By assumption, for every recovery map $R_{B\to BC}$ that satisfies (6.8) we have $f(R_{B\to BC})>0$ and hence *δ*∈(0,1]. If we insert any such recovery map $R_{B\to BC}$ into (6.8), this gives

6.9 $$1-F(\rho_{ABC},R_{B\to BC}(\rho_{AB}))+\frac{\delta }{p}-\delta \leq \frac{\ln(2)}{2}I{\left(A:C|B\right)}_{\rho },$$

which cannot be valid for sufficiently small *p*. To see this, we note that (6.9) can be rewritten as

6.10 $$p\geq \frac{\delta }{(\ln(2)/2)I{\left(A:C|B\right)}_{\rho }+\delta +F(\rho_{ABC},R_{B\to BC}(\rho_{AB}))-1},$$

since *C* is assumed to be a finite-dimensional system and as such $I{\left(A:C|B\right)}_{\rho }<\infty$. This contradicts our assumption that every recovery map that satisfies (6.8) does not leave $\rho_{ABC}^{0}$ invariant. Since by [6] for any *p*∈(0,1] there exists a recovery map $R_{B\to BC}$ of the form (6.3) that satisfies (6.8) we conclude that there exists a recovery map $R_{B\to BC}$ of the form (6.3) that satisfies (6.8) and leaves $\rho_{ABC}^{0}$ invariant. We note that for recovery maps that leave $\rho_{ABC}^{0}$ invariant, (6.8) simplifies to (6.4) for all *p*. Thus, there exists a recovery map $R_{B\to BC}$ of the form (6.3) satisfying (6.4) that leaves $\rho_{ABC}^{0}$ invariant, i.e. $R_{B\to BC}(\rho_{AB}^{0})=\rho_{ABC}^{0}$. Since $\rho_{ABC}^{0}:=\rho_{A}⊗\rho_{BC}$ is a Markov chain with marginal $\rho_{BC}^{0}=\rho_{BC}$, the condition $R_{B\to BC}(\rho_{AB}^{0})=\rho_{ABC}^{0}$ implies that $R_{B\to BC}(\rho_{B})=\rho_{BC}$.

We have thus shown that there exists a recovery map $R_{B\to BC}$ that satisfies (6.4) and fulfils

6.11 $$R_{B\to BC}(\rho_{B})=V_{BC}\rho_{BC}^{1/2}(U_{B}U_{B}^{†}⊗{\mathrm{id}}_{C})\rho_{BC}^{1/2}V_{BC}^{†}=\rho_{BC}.$$

Using the fact that $R_{B\to BC}$ is trace preserving shows that

6.12 $${\mathrm{id}}_{B}={\mathrm{tr}}_{C}(U_{B}^{†}\rho_{B}^{-1/2}\rho_{BC}^{1/2}V_{BC}V_{BC}^{†}\rho_{BC}^{1/2}\rho_{B}^{-1/2}U_{B})=U_{B}^{†}\rho_{B}^{-1/2}\rho_{B}\rho_{B}^{-1/2}U_{B}=U_{B}^{†}U_{B}.$$

This simplifies (6.11) to $V_{BC}\rho_{BC}V_{BC}^{†}=\rho_{BC}$, i.e. *V*_*BC*_ and *ρ*_*BC*_ commute which concludes the proof. ▪

Lemma 6.1 implies that the mapping (6.3) can be written as

6.13 $$X_{B}\mapsto \rho_{BC}^{1/2}W_{BC}(\rho_{B}^{-1/2}X_{B}\rho_{B}^{-1/2}⊗{\mathrm{id}}_{C})W_{BC}^{†}\rho_{BC}^{1/2},$$

with *W*_*BC*_=*V*_*BC*_*U*_*B*_⊗id_*C*_ which is a unitary as *V*_*BC*_ and *U*_*B*_ are unitaries. Furthermore, *W*_*BC*_ is such that (6.13) is trace-preserving.

### (b) Step 2. Structure of a universal recovery map for fixed *A* system

In this step, we show that the recovery map satisfying (6.1) of the form (6.2), whose existence has been established in Step 1, can be made universal without sacrificing the (partial) knowledge about its structure. The idea is to apply proposition 4.1 for the function family

6.14 $$\begin{array}{rl} & {\tilde{\Delta }}_{R}(\rho ):\;D(A⊗B⊗C)\to R\cup \{-\infty \}\\ & \;\rho_{ABC}\mapsto F(\rho_{ABC},R_{B\to BC}(\rho_{AB}))-1+\frac{\ln(2)}{2}I{\left(A:C|B\right)}_{\rho }. \end{array}$$

We therefore need to verify that the assumptions of proposition 4.1 are fulfilled. This is done by the following lemma. We first note that since *C* is finite-dimensional this implies that ${\tilde{\Delta }}_{R}(\rho )<\infty$ for all *ρ*∈*D*(*A*⊗*B*⊗*C*).

**Lemma 6.2**

*Let A be a separable and B and C finite-dimensional Hilbert spaces. The function family* ${\tilde{\Delta }}_{R}(\cdot )$ *defined by* (6.14) *satisfies properties* (i)–(iv).

**Proof.**

We start by showing that ${\tilde{\Delta }}_{R}(\cdot )$ satisfies property (i). For $\rho_{\hat{A}ABC}^{p}$ as defined in (4.4), we claim

6.15 $$F\left(\rho_{\hat{A}ABC}^{p},R_{B\to BC}(\rho_{\hat{A}AB}^{p})\right)=(1-p)F\left(\rho_{ABC}^{0},R_{B\to BC}(\rho_{AB}^{0})\right)+pF\left(\rho_{ABC},R_{B\to BC}(\rho_{AB})\right).$$

The density operator $R_{B\to BC}(\rho_{\hat{A}AB}^{p})$ can be written as

6.16 $$R_{B\to BC}(\rho_{\hat{A}AB}^{p})=(1-p)|0\rangle \langle 0{|}_{\hat{A}}⊗R_{B\to BC}(\rho_{AB}^{0})+p|1\rangle \langle 1{|}_{\hat{A}}⊗R_{B\to BC}(\rho_{AB}).$$

The relevant density operators thus satisfy the orthogonality conditions for equality in lemma 8.1 given in the electronic supplementary material, from which (6.15) follows. Furthermore, as explained in the proof of lemma 4.2 we have

6.17 $$I{\left(\hat{A}A:C|B\right)}_{\rho^{p}}=(1-p)I{\left(A:C|B\right)}_{\rho^{0}}+pI{\left(A:C|B\right)}_{\rho }.$$

Equations (6.15) and (6.17) imply that

6.18 $${\tilde{\Delta }}_{R}(\rho^{p})=(1-p){\tilde{\Delta }}_{R}(\rho^{0})+p{\tilde{\Delta }}_{R}(\rho ).$$

We next verify that ${\tilde{\Delta }}_{R}(\cdot )$ fulfils property (ii). Let $R_{B\to BC},R_{B\to BC}^{\prime }\in \mathrm{TPCP}(B,B⊗C)$, *α*∈[0,1] and ${\bar{R}}_{B\to BC}=\alpha R_{B\to BC}+(1-\alpha )R_{B\to BC}^{\prime }$. A specific property of the fidelity stated in lemma 8.1 in the electronic supplementary material implies that for any state *ρ*_*ABC*_ on *A*⊗*B*⊗*C*

6.19 $$\begin{array}{rl} & F(\rho_{ABC},{\bar{R}}_{B\to BC}(\rho_{AB}))=F(\rho_{ABC},\alpha R_{B\to BC}(\rho_{AB})+(1-\alpha )R_{B\to BC}^{\prime }(\rho_{AB}))\\ & \;\geq \alpha F(\rho_{ABC},R_{B\to BC}(\rho_{AB}))+(1-\alpha )F(\rho_{ABC},R_{B\to BC}^{\prime }(\rho_{AB})), \end{array}$$

and, hence, by the definition of ${\tilde{\Delta }}_{R}(\cdot )$

6.20 $${\tilde{\Delta }}_{\bar{R}}(\rho )\geq \alpha {\tilde{\Delta }}_{R}(\rho )+(1-\alpha ){\tilde{\Delta }}_{R^{\prime }}(\rho ).$$

The function $\rho \mapsto {\tilde{\Delta }}_{R}(\rho )$ is continuous which clearly implies property (iii). To see this, recall that by the Alicki–Fannes inequality *ρ*↦*I*(*A*:*C*|*B*)_*ρ*_ is continuous for a finite-dimensional *C* system [35]. Furthermore, since $\rho_{AB}\mapsto R_{BC}(\rho_{AB})$ is continuous (see lemma 10.5 in the electronic supplementary material), the continuity of the fidelity [12,13] implies that $\rho_{ABC}\mapsto F(\rho_{ABC},R_{B\to BC}(\rho_{AB}))$ is continuous, which then establishes property (iii).

Finally, it remains to show that ${\tilde{\Delta }}_{R}(\cdot )$ satisfies property (iv), which however follows directly by lemma 10.4 given in the electronic supplementary material. ▪

Let P⊆TPCP(B,B⊗C) be the convex hull of the set of trace-preserving completely positive mappings from the *B* to the *B*⊗*C* system that are of the form (6.2). We note that the elements of P are mappings of the form (2.4), since a convex combination of unitary mappings are unital and a convex combination of trace-preserving maps remains trace-preserving. Proposition 4.1, which is applicable as shown in lemma 6.2 together with Step 1 therefore proves the assertion for a fixed finite-dimensional *A* system.

### (c) Step 3. Independence from the *A* system

Let S be the set of all density operators on $\bar{A}⊗B⊗C$ with a fixed marginal *ρ*_*BC*_ on *B*⊗*C*, where *B* and *C* are finite-dimensional Hilbert spaces and $\bar{A}$ is the infinite-dimensional Hilbert space ℓ^2^ of square summable sequences.

We note that the set of trace-preserving completely positive maps of the form (2.4) on finite-dimensional systems is compact, which follows by remark 10.3 (see the electronic supplementary material) together with the fact that the intersection of a compact set and a closed set is compact. Hence, using lemma 6.2 (in particular properties (iii) and (iv)) and the result from Step 2, the same argument as in Step 4 of §4 can be applied to conclude the existence of a recovery map $R_{B\to BC}$ of the form (2.4) such that ${\tilde{\Delta }}_{R}(S)\geq 0$.

As every separable Hilbert space *A* can isometrically embedded into $\bar{A}$ [21], Theorem II.7 and since ${\tilde{\Delta }}_{\bar{R}}$ is invariant under isometries applied on the extension space *A*, we can conclude that the recovery map $R_{B\to BC}$ remains valid for any separable extension space *A*. This proves the statement of corollary 2.4 for finite-dimensional *B* and *C* systems.

## 7. Discussion

Our main result is that for any density operator *ρ*_*BC*_ on *B*⊗*C* there exists a recovery map $R_{B\to BC}$ such that the distance between *any* extension *ρ*_*ABC*_ of *ρ*_*BC*_ acting on *A*⊗*B*⊗*C* and $R_{B\to BC}(\rho_{AB})$ is bounded from above by the conditional mutual information *I*(*A*:*C*|*B*)_*ρ*_. It is natural to ask whether such a map can be described as a simple and explicit function of *ρ*_*BC*_. In fact, it was conjectured in [5,11] that (1.2) holds for a very simple choice of map, namely

7.1 $$T_{B\to BC}:X_{B}\mapsto \rho_{BC}^{1/2}(\rho_{B}^{-1/2}X_{B}\rho_{B}^{-1/2}⊗{\mathrm{id}}_{C})\rho_{BC}^{1/2},$$

called the *transpose map* or *Petz recovery map*. This conjecture, if correct, would have important consequences in obtaining remainder terms for the monotonicity of the relative entropy [19]. As discussed in the Introduction, if *ρ*_*ABC*_ is such that it is a (perfect) quantum Markov chain or the *B* system is classical, the claim of the conjecture is known to hold.

One possible approach to prove a result of this form would be to start from the result (1.2) for an unknown recovery map and then show that the transpose map $T_{B\to BC}$ cannot be much worse than any other recovery map. In fact, a theorem of Barnum & Knill [48] directly implies that when *ρ*_*ABC*_ is pure, we have

7.2 $$F(\rho_{ABC},T_{B\to BC}(\rho_{AB}))\leq F{\left(A;C|B\right)}_{\rho }\leq \sqrt{F(\rho_{ABC},T_{B\to BC}(\rho_{AB}))}.$$

This shows that, if *ρ*_*ABC*_ is pure, an inequality of the form (1.2), with the fidelity replaced by its square root, holds for the transpose map. In order to generalize this to all states, one might hope that (7.2) also holds for mixed states *ρ*_*ABC*_. However, this turns out to be wrong even when the state *ρ*_*ABC*_ is completely classical (see §13 in the electronic supplementary material for an example).

Another interesting question is whether the lower bound in terms of the measured relative entropy (2.2) can be improved to a relative entropy. Such an inequality is known to be false if we restrict the recovery map to be the transpose map (7.1) [9], but it might be true when we optimize over all recovery maps. It is worth noting that in case such an inequality holds for any *ρ*_*ABC*_ and a corresponding recovery map, then the argument presented in this work would imply that there exists a universal recovery map satisfying (2.2) with the relative entropy instead of the measured relative entropy. This can be seen by defining the function family $\rho \mapsto \Delta_{R}(\rho ):=I{\left(A:C|B\right)}_{\rho }-D(\rho_{ABC}∥R_{B\to BC}(\rho_{AB}))$. A linearity property of the relative entropy for orthogonal states (see lemma 9.2 in the electronic supplementary material), the convexity of the relative entropy [49], theorem 11.12 and the lower semicontinuity of the relative entropy [41], example 7.22 imply that $\Delta_{R}(\cdot )$ satisfies properties (i)–(iv). As a result, proposition 4.1 is applicable which can be used to prove the existence of a universal recovery map.

After the completion of this work, there was a series of works around finding improvements or alternative proofs for inequality (1.2). In [20], an alternative proof for (1.2) based on the Hadamard three-line theorem was discovered.^14^ After that yet another proof for (1.2) has been found which is based elementary properties of pinching maps and the operator logarithm [50]. Finally in [40] an *explicit* and universal recovery map has been determined that satisfies (1.2) based on Hirschman’s strengthening [51] of the Hadamard three-line theorem.

## Supplementary Material

Supplementary Information

## Footnotes

1 The fidelity of *ρ* and *σ* is defined as $F(\rho ,\sigma ):=∥\sqrt{\rho }\sqrt{\sigma }{∥}_{1}$.

2 We note that if *A*, *B* and *C* are finite-dimensional Hilbert spaces the supremum is achieved, since the set of recovery maps is compact (see remark 10.3 in the electronic supplementary material) and the fidelity is continuous in the input state (see [12,13] or lemma B.9 in [6]).

3 Without loss of generality, these can be assumed to be rank-one projectors.

4 This follows by the equidistribution theorem which is a special case of the strong ergodic theorem [21], §II.5 (see also [22]).

5 More precisely, we require that ∥*tr*_*G*_(*ρ*^*i*^)−*tr*_*G*_(*ρ*^*j*^)∥_1_=*o*(*n*^−2^).

6 Note that there exist different quantities that are called topological entanglement entropy (see also [34]).

7 For *n*=0, we have $\Delta_{R}(S)=\infty \geq 0$ for any R∈P since the infimum of an empty set is infinity.

8 The precise form of the sequence ${\left\{\xi^{b,c}\right\}}_{b,c\in N}$ is given in the proof (see equation (5.17)).

9 For *x*=0 the statement clearly holds. For $(0,1]\times [0,1]∋(x,y)\mapsto f(x,y):=x^{y}-x+y-1\in R$ we find by using the convexity of *y*↦*f*(*x*,*y*) that $\max_{x\in (0,1]}\max_{y\in [0,1]}f(x,y)=0$.

10 The precise form of the sequence ${\left\{\delta^{m}\right\}}_{m\in N}$ can be found in the proof (see equation (5.28)).

11 We note that the set of sub-normalized density operators on a Hilbert space is clearly closed. Since every Hilbert space is complete and as every closed subspace of a complete space is complete [42], ch. II, section 3.4, proposition 8 this implies that the set of sub-normalized density operators is complete.

12 Although corollary 2.4 does not immediately follow from theorem 2.1 it is justified to term it as such, as it follows by the same proof technique that is used to derive theorem 2.1 (in particular it makes use of proposition 4.1).

13 This set is bounded as the set of trace-preserving completely positive maps from *B* to *B*⊗*C* is bounded (see remark 10.3 in the electronic supplementary material). Furthermore, this set is closed since the set of trace-preserving completely positive maps from *B* to *B*⊗*C* is closed (see remark 10.3 in the electronic supplementary material) and the mapping $R_{B\to BC}\mapsto F(\rho_{ABC},R_{B\to BC}(\rho_{AB}))$ is continuous for all states *ρ*_*ABC*_ (see lemma 10.4 in the electronic supplementary material). The Heine–Borel theorem then implies compactness.

14 The result in [20] is more general than (1.2) as it proves a remainder term for the monotonicity of the relative entropy which implies (1.2) as a special case (see [20] for more details).

## Data accessibility

This work does not have any experimental data.
