# Supplementary material for: Universal recovery map for approximate Markov chains
Source: Proc Math Phys Eng Sci. 2016 Feb;472(2186):20150623. doi: 10.1098/rspa.2015.0623 (PMC4841654; doi:10.1098/rspa.2015.0623)
Supplement: Supplementary Information [file rspa20150623supp1.pdf]

Supplementary information

In this supplementary information we provide proofs for some technical statements that are used in the main document.

8 General facts about the fidelity

The following lemma states a standard concavity property of the fidelity which is presented here for completeness and since we are interested in the case where equality holds.

Lemma 8.1. *For any density operators ρ, ρ', σ, and σ', and for any $p \in [0,1]$ we have*

$$F\big(p\rho + (1-p)\rho', p\sigma + (1-p)\sigma'\big) \geq pF(\rho,\sigma) + (1-p)F(\rho',\sigma') \ , \tag{S.1}$$

with equality if both of ρ and σ are orthogonal to both of ρ' and σ'.

Proof. Note first that for any two normalized and mutually orthogonal vectors $|0\rangle$ and $|1\rangle$ in an ancilla space, we have

$$F\big(p\rho+(1-p)\rho', p\sigma+(1-p)\sigma'\big) \geq F\big(p\rho\otimes|0\rangle\langle 0|+(1-p)\rho'\otimes|1\rangle\langle 1|, p\sigma\otimes|0\rangle\langle 0| + (1-p)\sigma'\otimes|1\rangle\langle 1|\big) \ , \tag{S.2}$$

because of the monotonicity of the fidelity under the partial trace. Furthermore, if both of ρ and σ are orthogonal to both of ρ' and σ' then there exists a trace-preserving completely positive map that generates the corresponding state $|0\rangle$ or $|1\rangle$ of the ancilla system. This implies that, in this case, the inequality also holds in the other direction. It therefore suffices to prove (S.1) with ρ and σ replaced by $\rho \otimes |0\rangle\langle 0|$ and $\sigma \otimes |0\rangle\langle 0|$, and with ρ' and σ' replaced by $\rho' \otimes |1\rangle\langle 1|$ and $\sigma' \otimes |1\rangle\langle 1|$, respectively. In other words, it remains to show that, for the case where ρ and σ are orthogonal to ρ' and σ', (S.1) holds with equality, i.e.,

$$F(\bar{\rho},\bar{\sigma}) = pF(\rho,\sigma) + (1-p)F(\rho',\sigma') \ , \tag{S.3}$$

where $\bar{\rho} = p\rho + (1-p)\rho'$ and $\bar{\sigma} = p\sigma + (1-p)\sigma'$.

For this, let $|\phi\rangle$, $|\phi'\rangle$, $|\psi\rangle$, and $|\psi'\rangle$ be purifications of ρ, ρ', σ, and σ', respectively, such that $F(\rho,\sigma) = \langle\phi|\psi\rangle$ and $F(\rho',\sigma') = \langle\phi'|\psi'\rangle$. It is easy to verify that

$$|\bar{\phi}\rangle = \sqrt{p}|\phi\rangle \otimes |0\rangle + \sqrt{1-p}|\phi'\rangle \otimes |1\rangle \quad \text{and} \quad |\bar{\psi}\rangle = \sqrt{p}|\psi\rangle \otimes |0\rangle + \sqrt{1-p}|\psi'\rangle \otimes |1\rangle \tag{S.4}$$

are purifications of $\bar{\rho}$ and of $\bar{\sigma}$, respectively. Hence,

$$pF(\rho,\sigma) + (1-p)F(\rho',\sigma') = p\langle\phi|\psi\rangle + (1-p)\langle\phi'|\psi'\rangle = \langle\bar{\phi}|\bar{\psi}\rangle \leq F(\bar{\rho},\bar{\sigma}) \ , \tag{S.5}$$

which proves one direction of (S.3).

To prove the other direction, let π be the projector onto the joint support of ρ and σ, i.e., $\pi\rho = \rho$ and $\pi\sigma = \sigma$. Similarly, let π' be the projector onto the joint support of ρ' and σ', i.e.,. $\pi'\rho' = \rho'$ and $\pi'\sigma' = \sigma'$. By the condition that ρ and σ are orthogonal to ρ' and σ', the two projectors must be orthogonal, i.e., $\pi\pi' = 0$. Furthermore, let $|\bar{\phi}\rangle$ be a purification of $\bar{\rho}$ and let $|\bar{\psi}\rangle$ be a purification of $\bar{\sigma}$ such that $F(\bar{\rho},\bar{\sigma}) = \langle\bar{\phi}|\bar{\psi}\rangle$. Because

$$p\rho = \pi\bar{\rho}\pi \quad \text{and} \quad (1-p)\rho' = \pi'\bar{\rho}\pi' \tag{S.6}$$

$\pi|\bar{\phi}\rangle$ and $\pi'|\bar{\phi}\rangle$ are purifications of $p\rho$ and $(1-p)\rho'$, respectively. Similarly, $\pi|\bar{\psi}\rangle$ and $\pi'|\bar{\psi}\rangle$ are purifications of $p\sigma$ and $(1-p)\sigma'$, respectively. Hence, we have

$$\begin{aligned} F(\bar{\rho},\bar{\sigma}) = \langle\bar{\phi}|\bar{\psi}\rangle = \langle\bar{\phi}|\pi|\bar{\psi}\rangle + \langle\bar{\phi}|\pi'|\bar{\psi}\rangle &\leq F\big(p\rho, p\sigma\big) + F\big((1-p)\rho', (1-p)\sigma'\big) \\ &= pF(\rho,\sigma) + (1-p)F(\rho',\sigma') \ . \end{aligned} \tag{S.7}$$

This proves the other direction of (S.3) and thus concludes the proof. □

The following lemma generalizes the Fuchs-van de Graaf inequality which has been proven for states to non-negative operators. The result is standard and stated here for completeness.

Lemma 8.2. *For any two non-negative operators ρ and σ with $\operatorname{tr}(\rho) \geq \operatorname{tr}(\sigma)$, the trace norm of their difference is bounded from above by*

$$\|\rho - \sigma\|_1 \leq 2\sqrt{\operatorname{tr}(\rho)^2 - F(\rho,\sigma)^2} \ . \tag{S.8}$$

Proof. Let ω be a non-negative operator with $\operatorname{tr}(\omega) = \operatorname{tr}(\rho) - \operatorname{tr}(\sigma)$, whose support is orthogonal to the support of both ρ and σ, and define $\sigma' = \sigma + \omega$. Then $\operatorname{tr}(\rho) = \operatorname{tr}(\sigma')$ and

$$\|\rho - \sigma\|_1 = \|\rho - \sigma'\|_1 \qquad \text{and} \qquad F(\rho,\sigma) = F(\rho,\sigma') \ . \tag{S.9}$$

It therefore suffices to show that the claim holds for operators with $\operatorname{tr}(\rho) = \operatorname{tr}(\sigma) = c \in \mathbb{R}^+$. Furthermore for $c > 0$, defining $\bar{\rho} = \rho/c$ and $\bar{\sigma} = \sigma/c$ and noting that

$$\|\rho - \sigma\|_1 = c\,\|\bar{\rho} - \bar{\sigma}\|_1 \qquad \text{and} \qquad F(\rho,\sigma) = cF(\bar{\rho},\bar{\sigma}) \ , \tag{S.10}$$

it suffices to verify that the claim holds for $\operatorname{tr}(\rho) = \operatorname{tr}(\sigma) = 1$ which follows by the Fuchs-van de Graaf inequality [FvdG99]. □

9 General facts about the measured relative entropy

Definition 9.1. The *measured relative entropy* between density operators ρ and σ is defined as the supremum of the relative entropy with measured inputs over all POVMs $\mathcal{M} = \{M_x\}$, i.e.,

$$D_{\mathbb{M}}(\rho\|\sigma) = \sup\Big\{D(\mathcal{M}(\rho)\|\mathcal{M}(\sigma)) : \mathcal{M}(\rho) = \sum_x \operatorname{tr}(\rho M_x)|x\rangle\langle x| \text{ with } \sum_x M_x = \mathrm{id}\Big\} \ , \tag{S.11}$$

where $\{|x\rangle\}$ is a finite set of orthonormal vectors.

This quantity was studied in [HP91, Hay01] where it was shown that $\frac{1}{n}D_{\mathbb{M}}(\rho^{\otimes n}\|\sigma^{\otimes n})$ converges to the relative entropy $D(\rho\|\sigma) := \operatorname{tr}(\rho(\log\rho - \log\sigma))$.

Lemma 9.2. *Let ρ, ρ', σ, and σ' be density operators such that both ρ and σ are orthogonal to both ρ' and σ'. For any $p \in [0,1]$ we have*

$$D\big(p\rho + (1-p)\rho' \,\|\, p\sigma + (1-p)\sigma'\big) = pD(\rho\|\sigma) + (1-p)D(\rho'\|\sigma') \ . \tag{S.12}$$

Proof. By the orthogonality of ρ and ρ' (respectively σ and σ') we have

$$\log\big(p\rho + (1-p)\rho'\big) = \log(p\rho) + \log\big((1-p)\rho'\big) = \log(p) + \log(1-p) + \log(\rho) + \log(\rho') \tag{S.13}$$

and $\rho\log\rho' = 0$. Thus by definition of the relative entropy we obtain the desired statement. □

Lemma 9.3. *Let ρ, ρ', σ, and σ' be density operators such that both ρ and σ are orthogonal to both ρ' and σ'. For any $p \in [0,1]$ we have*

$$D_{\mathbb{M}}\big(p\rho + (1-p)\rho' \,\|\, p\sigma + (1-p)\sigma'\big) = pD_{\mathbb{M}}(\rho\|\sigma) + (1-p)D_{\mathbb{M}}(\rho'\|\sigma') \ . \tag{S.14}$$

Proof. Let $\mathcal{M} = \{M_x\}$, $\mathcal{M}' = \{M'_y\}$ be measurements and define the POVM on $\mathcal{N}$ whose elements are given by $\{M_x\}_x \cup \{M'_y\}_y$. Then we can write

$$\mathcal{N}\big(p\rho + (1-p)\rho'\big) = p\sum_x \operatorname{tr}(M_x\rho)|x\rangle\langle x| + (1-p)\sum_y \operatorname{tr}(M'_y\rho')|y\rangle\langle y| \ . \tag{S.15}$$

As a result using Lemma 9.2,

$$\begin{aligned}&D_{\mathbb{M}}\big(p\rho+(1-p)\rho' \,\|\, p\sigma+(1-p)\sigma'\big) \geq D\Big(\mathcal{N}\big(p\rho+(1-p)\rho'\big) \,\Big\|\, \mathcal{N}\big(p\sigma+(1-p)\sigma'\big)\Big)\\ &\quad= pD\Big(\sum_x \operatorname{tr}(M_x\rho)|x\rangle\langle x| \Big\| \sum_x \operatorname{tr}(M_x\sigma)|x\rangle\langle x|\Big) + (1-p)D\Big(\sum_y \operatorname{tr}(M'_y\rho')|y\rangle\langle y| \Big\| \sum_y \operatorname{tr}(M'_y\sigma')|y\rangle\langle y|\Big) \ .\end{aligned} \tag{S.16}$$

As this inequality is valid for any measurements $\mathcal{M}$ and $\mathcal{M}'$, taking the supremum over such measurements gives

$$D_{\mathbb{M}}\big(p\rho+(1-p)\rho' \,\|\, p\sigma+(1-p)\sigma'\big) \geq pD_{\mathbb{M}}(\rho\|\sigma) + (1-p)D_{\mathbb{M}}(\rho'\|\sigma') \ . \tag{S.17}$$

For the other direction, consider a measurement $\mathcal{M} = \{M_x\}$. We can write

$$\mathcal{M}\big(p\rho+(1-p)\rho'\big) = \sum_x p \operatorname{tr}(M_x\rho)|x\rangle\langle x| + (1-p)\operatorname{tr}(M_x\rho')|x\rangle\langle x| \ . \tag{S.18}$$

Combining this with the joint convexity of the relative entropy [NC00, Theorem 11.12], we get

$$\begin{aligned}&D_{\mathbb{M}}\big(p\rho+(1-p)\rho' \,\|\, p\sigma+(1-p)\sigma'\big) = D\Big(\mathcal{M}\big(p\rho+(1-p)\rho'\big) \Big\| \mathcal{M}\big(p\sigma+(1-p)\sigma'\big)\Big)\\ &\leq p\,D\Big(\sum_x \operatorname{tr}(M_x\rho)|x\rangle\langle x| \Big\| \sum_x \operatorname{tr}(M_x\sigma)|x\rangle\langle x|\Big) + (1-p)\,D\Big(\sum_x \operatorname{tr}(M_x\rho')|x\rangle\langle x| \Big\| \sum_x \operatorname{tr}(M_x\sigma')|x\rangle\langle x|\Big)\\ &\leq pD_{\mathbb{M}}(\rho\|\sigma) + (1-p)D_{\mathbb{M}}(\rho'\|\sigma') \ .\end{aligned} \tag{S.19}$$

□

Lemma 9.4. *For density operators ρ, σ, and σ' and $p \in [0,1]$ the measured relative entropy satisfies*

$$D_{\mathbb{M}}\big(\rho\|p\sigma+(1-p)\sigma'\big) \leq p\,D_{\mathbb{M}}(\rho\|\sigma) + (1-p)\,D_{\mathbb{M}}(\rho\|\sigma') \ . \tag{S.20}$$

Proof. For any measurement $\mathcal{M}$,

$$\begin{aligned}D\big(\mathcal{M}(\rho)\|\mathcal{M}(p\sigma+(1-p)\sigma')\big) &= D\big(\mathcal{M}(\rho)\|\,p\,\mathcal{M}(\sigma)+(1-p)\mathcal{M}(\sigma')\big)\\ &\leq p\,D\big(\mathcal{M}(\rho)\|\mathcal{M}(\sigma)\big) + (1-p)\,D\big(\mathcal{M}(\rho)\|\mathcal{M}(\sigma')\big)\\ &\leq pD_{\mathbb{M}}(\rho\|\sigma) + (1-p)D_{\mathbb{M}}(\rho\|\sigma') \ ,\end{aligned} \tag{S.21}$$

where the first inequality step uses the convexity of the relative entropy [NC00, Theorem 11.12]. Taking the supremum over $\mathcal{M}$, we get the desired result. □

10 Basic topological facts

For completeness we state here some standard topological facts about density operators and trace-preserving completely positive maps.

Lemma 10.1. *Let $\alpha \in \mathbb{R}^+$. The space of non-negative operators on a finite-dimensional Hilbert space E with trace smaller or equal to α (respectively equal to α) is compact.*

Proof. Let $\mathrm{D}'(E) := \{\rho \in \mathrm{Pos}(E) : \operatorname{tr}(\rho) \leq \alpha\}$ denote the set non-negative operators on E with trace not larger than one, where $\mathrm{Pos}(E)$ is the set of non-negative operators on E. Consider the ball $\mathcal{B} := \{e \in E : \|e\| \leq \alpha\}$ which is compact. The function $\mathcal{B} \ni e \mapsto f(e) = ee^\dagger \in \mathrm{D}'(E)$ is continuous and thus the set $f(\mathcal{B}) = \{ee^\dagger : e \in E, \|e\| \leq \alpha\}$ is compact, as continuous functions map compact sets to compact sets. By the spectral theorem it follows that $\mathrm{D}'(E) = \operatorname{conv} f(\mathcal{B})$. As the convex hull of every compact set is compact this proves the assertion. The same argumentation (by replacing the inequalities with equalities) proves that the set of non-negative operators on E with trace α is compact. □

Lemma 10.2. *Let E, G be finite-dimensional Hilbert spaces and let $\sigma_G \in \text{Pos}(G)$. The space of non-negative operators on $E \otimes G$ with a marginal on G smaller or equal to σ_G (respectively equal to σ_G) is compact.*

Proof. Let $\sigma_G \in \text{Pos}(G)$. By Lemma 10.1, the set of non-negative operators on $E \otimes G$ with trace not larger than $\alpha \in \mathbb{R}^+$ is compact. The set $\{X \in E \otimes G : \text{tr}_E(X) \leq \rho_G\}$ is closed. The intersection of a compact set and a closed set is compact which implies that $\{X \in \text{Pos}(E \otimes G) : \text{tr}_E(X) \leq \rho_G\}$ is compact. Since the set $\{X \in E \otimes G : \text{tr}_E(X) = \rho_G\}$ is closed the same argumentation shows that $\{X \in \text{Pos}(E \otimes G) : \text{tr}_E(X) = \rho_G\}$ is compact. □

Remark 10.3. Let E and G be two finite-dimensional Hilbert spaces. The space of trace-non-increasing (respectively trace-preserving) completely positive maps from E to G is compact. To see this note that Lemma 10.2 implies that the set $\mathcal{F} := \{X \in \text{Pos}(E \otimes G) : \text{tr}_G(X) \leq \text{id}_E\}$ is compact. By the Choi-Jamiolkowski representation $\mathcal{F}$ is however isomorphic to the set of all trace-non-increasing completely positive maps from E to G. The same argumentation applied to the set $\mathcal{F} := \{X \in \text{Pos}(E \otimes G) : \text{tr}_G(X) = \text{id}_E\}$ shows that the set of trace-preserving completely positive maps from E to G is compact.

Lemma 10.4. *Let G and K be finite-dimensional Hilbert spaces and let $\sigma_{EGK} \in \text{D}(E \otimes G \otimes K)$. The mapping $\text{TPCP}(G, G \otimes K) \ni \mathcal{R} \mapsto F(\sigma_{EGK}, \mathcal{R}_{G \to GK}(\sigma_{EGK})) \in [0,1]$ is continuous.*

Proof. This follows directly from the continuity of $\mathcal{R} \mapsto \mathcal{R}_{G \to GK}(\sigma_{EG})$ and the continuity of the fidelity (see, e.g., Lemma B.9 of [FR14]). □

Lemma 10.5. *Let E, G, and K be separable Hilbert spaces and $\mathcal{R} \in \text{TPCP}(G, K)$. Then the mapping $\text{D}(E \otimes G) \ni X \mapsto \mathcal{I}_E \otimes \mathcal{R}_{G \to K}(X_{EG}) \in \text{D}(E \otimes K)$ is continuous.*

Proof. As the map is linear it suffices to show that it is bounded. For that we can decompose $X = P - N$ with P and N orthogonal non-negative operators. Then we have

$$\|\mathcal{I}_E \otimes \mathcal{R}_{G \to K}(X)\|_1 \leq \|\mathcal{I}_E \otimes \mathcal{R}_{G \to K}(P)\|_1 + \|\mathcal{I}_E \otimes \mathcal{R}_{G \to K}(N)\|_1 = \text{tr}(P) + \text{tr}(N) = \|X\|_1 \ . \quad \text{(S.22)}$$

□

11 Touching sets lemma

We prove here a basic fact that is used in the proof of Theorem 2.1.

Lemma 11.1. *Let K_0 and K_1 be two sets such that $K_0 \cup K_1 = [0,1]$ and $0 \in K_0$, $1 \in K_1$. Then for any $\delta > 0$ there exists $u \in K_0$ and $v \in K_1$ such that $0 \leq v - u \leq \delta$.*

Proof. We define $\mu := \inf K_1$ and distinguish between the two cases $\mu \in K_0$ and $\mu \notin K_0$.

If $\mu \in K_0$, it suffices to show that for any $\delta > 0$ we have $[\mu, \mu + \delta] \cap K_1 \neq \emptyset$, since by choosing $u = \mu$ this implies that $u \in K_0$ and that there exists a $v \in [\mu, \mu + \delta]$ such that $v \in K_1$. By contradiction, we assume that $[\mu, \mu + \delta] \cap K_1 = \emptyset$. This implies that either $\inf K_1 < \mu$ or $\inf K_1 \geq \mu + \delta$, which contradicts $\mu := \inf K_1$.

If $\mu \notin K_0$ it suffices to show that for any $\delta > 0$ we have $[\mu - \delta, \mu] \cap K_0 \neq \emptyset$, since by choosing $v = \mu$ this ensures that $v \in K_1$ and that there exists a $u \in [\mu - \delta, \mu]$ such that $u \in K_0$. Assume by contradiction that $[\mu - \delta, \mu] \cap K_0 = \emptyset$, which implies that $[\mu - \delta, \mu] \subset K_1$. This however contradicts $\mu := \inf K_1$.

□

12 Properties of projected states

We first prove variant of the *gentle measurement lemma* [Win99], which is used repeatedly in the proof of Theorem 2.1.

Lemma 12.1. *Let E and G be separable Hilbert spaces and let Π_G be a finite-rank projector on G. For any non-negative operator σ_{EG} on $E \otimes G$ we have*

$$F\left(\sigma_{EG}, \frac{(\mathrm{id}_E \otimes \Pi_G)\sigma_{EG}(\mathrm{id}_E \otimes \Pi_G)}{\mathrm{tr}\big((\mathrm{id}_E \otimes \Pi_G)\sigma_{EG}\big)}\right)^2 \geq \mathrm{tr}(\Pi_G \sigma_{EG}) \tag{S.23}$$

and

$$F\big(\sigma_{EG}, (\mathrm{id}_E \otimes \Pi_G)\sigma_{EG}(\mathrm{id}_E \otimes \Pi_G)\big) \geq \mathrm{tr}(\Pi_G \sigma_{EG}) \ . \tag{S.24}$$

Proof. Let $|\psi\rangle$ be a purification of σ_{EG} then by Uhlmann's theorem [Uhl76] we find

$$F\left(\sigma_{EG}, \frac{(\mathrm{id}_E \otimes \Pi_G)\sigma_{EG}(\mathrm{id}_E \otimes \Pi_G)}{\mathrm{tr}\big((\mathrm{id}_E \otimes \Pi_G)\sigma_{EG}\big)}\right)^2 \geq \frac{(\langle\psi|\Pi_G|\psi\rangle)^2}{\mathrm{tr}\big((\mathrm{id}_E \otimes \Pi_G)\sigma_{EG}\big)} = \mathrm{tr}(\Pi_G \sigma_{EG}) \tag{S.25}$$

and

$$F\big(\sigma_{EG}, (\mathrm{id}_E \otimes \Pi_G)\sigma_{EG}(\mathrm{id}_E \otimes \Pi_G)\big)^2 \geq (\langle\psi|\Pi_G|\psi\rangle)^2 = \mathrm{tr}(\Pi_G \sigma_{EG})^2 \ . \tag{S.26}$$

□

We next prove a basic statement about converging projectors that is used several times in the proof of Theorem 2.1.

Lemma 12.2. *Let E be a separable Hilbert space and let $\{\Pi_E^e\}_{e \in E}$ be a sequence of finite-rank projectors on E which converges to id_E with respect to the weak operator topology. Then for any density operator σ_E on E we have $\lim_{e\to\infty} \mathrm{tr}(\Pi_E^e \sigma_E) = \mathrm{tr}(\sigma_E)$.*

Proof. By assumption the Hilbert space E is separable which implies that any state σ_E can be written as $\sigma_E = \sum_i p_i |x_i\rangle\langle x_i|$, where $p_i \geq 0$, $\sum_i p_i = 1$ and $\{|x_i\rangle\}_i$ is an orthonormal basis on E. As the sequence $\{\Pi_E^e\}_{e\in\mathbb{N}}$ weakly converges to id_E, we find

$$\lim_{e\to\infty} \mathrm{tr}(\Pi_E^e \sigma_E) = \lim_{e\to\infty} \sum_i p_i \langle x_i|\Pi_E^e|x_i\rangle = \sum_i p_i \lim_{e\to\infty} \langle x_i|\Pi_E^e|x_i\rangle = \sum_i p_i \langle x_i|\mathrm{id}_E|x_i\rangle = \mathrm{tr}(\sigma_E) \ , \tag{S.27}$$

where the second step uses dominated convergence that is applicable since $|\langle x_i|\Pi_E^e|x_i\rangle| \leq |\langle x_i|\mathrm{id}_E|x_i\rangle|$ for all $e \in \mathbb{N}$. □

Let E and G be separable Hilbert spaces and let $\mathcal{S}$ denote the set of bipartite density operators on $E \otimes G$ with a fixed marginal σ_G on G. Let $\{\Pi_E^e\}_{e\in\mathbb{N}}$ be a sequence of projectors with rank e that weakly converge to id_E and $\mathcal{S}^e$ be the set of bipartite states on $E \otimes G$ whose marginal on E is contained in the support of Π_E^e and whose marginal on G is identical to σ_G.

Lemma 12.3. *For every $\sigma_{EG} \in \mathcal{S}$ there exists a sequence $\{\sigma_{EG}^e\}_{e\in\mathbb{N}}$ with $\sigma_{EG}^e \in \mathcal{S}^e$ that converges to σ_{EG} with respect to the trace norm.*

Proof. For $\sigma_{EG} \in \mathcal{S}$, let

$$\bar{\sigma}_{EG}^e := \frac{(\Pi_E^e \otimes \mathrm{id}_G)\sigma_{EG}(\Pi_E^e \otimes \mathrm{id}_G)}{\mathrm{tr}\big((\Pi_E^e \otimes \mathrm{id}_G)\sigma_{EG}\big)} \ , \tag{S.28}$$

which has the desired support on E, however, $\bar{\sigma}^e_G \neq \sigma_G$ in general. This is fixed by considering

$$\sigma^e_{EG} := \mathrm{tr}\big((\Pi^e_E \otimes \mathrm{id}_G)\sigma_{EG}\big)\bar{\sigma}^e_{EG} + |0\rangle\langle 0|_E \otimes \mathrm{tr}_E\big((\Pi^{e\perp}_E \otimes \mathrm{id}_G)\sigma_{EG}(\Pi^{e\perp}_E \otimes \mathrm{id}_G)\big)_G \ , \tag{S.29}$$

where $|0\rangle_E$ is a normalized state on E. Since the partial trace on E is cyclic on E we obtain

$$\begin{aligned}\sigma^e_G = \mathrm{tr}_E(\sigma^e_{EG}) &= \mathrm{tr}_E\big((\Pi^e_E \otimes \mathrm{id}_G)\sigma_{EG}(\Pi^e_E \otimes \mathrm{id}_G)\big) + \mathrm{tr}_E\big((\Pi^{e\perp}_E \otimes \mathrm{id}_G)\sigma_{EG}(\Pi^{e\perp}_E \otimes \mathrm{id}_G)\big)\\ &= \mathrm{tr}_E\big((\Pi^e_E \otimes \mathrm{id}_G)\sigma_{EG}\big) + \mathrm{tr}_E\big((\Pi^{e\perp}_E \otimes \mathrm{id}_G)\sigma_{EG}\big) = \mathrm{tr}_E(\sigma_{EG}) = \sigma_G \ . \end{aligned}\tag{S.30}$$

By the multiplicativity of the trace norm under tensor products and since $\|A\|_1 = \mathrm{tr}(\sqrt{A^\dagger A})$, the triangle inequality implies that

$$\begin{aligned}\|\bar{\sigma}^e_{EG} - \sigma^e_{EG}\|_1 &\leq 1 - \mathrm{tr}\big((\Pi^e_E \otimes \mathrm{id}_G)\sigma_{EG}\big) + \big\|\mathrm{tr}_E\big((\Pi^{e\perp}_E \otimes \mathrm{id}_G)\sigma_{EG}(\Pi^{e\perp}_E \otimes \mathrm{id}_G)\big)\big\|_1\\ &= 1 - \mathrm{tr}\big((\Pi^e_E \otimes \mathrm{id}_G)\sigma_{EG}\big) + \mathrm{tr}\big((\Pi^{e\perp}_E \otimes \mathrm{id}_G)\sigma_{EG}\big) = 2\big(1 - \mathrm{tr}(\Pi^e_E\sigma_E)\big) \ . \end{aligned}\tag{S.31}$$

Lemma 12.2 now implies that $\lim_{e\to\infty} \mathrm{tr}(\Pi^e_E\sigma_E) = 1$. We note that the sequence $\{\bar{\sigma}^e_{EG}\}_{e\in\mathbb{N}}$ converges to σ_{EG} in the trace norm since by the Fuchs-van de Graaf inequality [FvdG99], Lemma 12.1 and Lemma 12.2

$$\lim_{e\to\infty} \|\sigma_{EG} - \bar{\sigma}^e_{EG}\|_1 \leq \lim_{e\to\infty} 2\sqrt{1 - F(\sigma_{EG}, \bar{\sigma}^e_{EG})^2} \leq \lim_{e\to\infty} 2\sqrt{1 - \mathrm{tr}(\Pi^e_E\sigma_E)} = 0 \ . \tag{S.32}$$

Combining this with (S.31) and the triangle inequality proves that $\{\sigma^e_{EG}\}_{e\in\mathbb{N}}$ converges to σ_{EG} in the trace norm. □

13 The transpose map is not square-root optimal

As discussed in Section 7 (see main document), for pure states ρ_{ABC} it is known [BK02] that

$$F(A;C|B)_\rho \leq \sqrt{F\big(\rho_{ABC}, \mathcal{T}_{B\to BC}(\rho_{AB})\big)} \tag{S.33}$$

holds for $\mathcal{T}_{B\to BC}$ the transpose map. In this appendix we show that (S.33) does not hold for all mixed states. Let $\dim A = \dim B = \dim C = 2$ and consider the state

$$\rho_{ABC} = \frac{1}{2}|0\rangle\langle 0|_A \otimes |0\rangle\langle 0|_B \otimes |0\rangle\langle 0|_C + \frac{1}{8}|1\rangle\langle 1|_A \otimes \mathrm{id}_{BC} \ . \tag{S.34}$$

The transpose map satisfies

$$\mathcal{T}_{B\to BC}(|0\rangle\langle 0|_B) = \frac{5}{6}|00\rangle\langle 00|_{BC} + \frac{1}{6}|01\rangle\langle 01|_{BC} \quad \text{and} \quad \mathcal{T}_{B\to BC}(|1\rangle\langle 1|_B) = \frac{1}{2}|10\rangle\langle 10|_{BC} + \frac{1}{2}|11\rangle\langle 11|_{BC} \ . \tag{S.35}$$

If we consider a recovery map $\mathcal{R}_{B\to BC}$ that is defined by

$$\mathcal{R}_{B\to BC}(|0\rangle\langle 0|_B) = |00\rangle\langle 00|_{BC} \quad \text{and} \quad \mathcal{R}_{B\to BC}(|1\rangle\langle 1|_B) = \frac{1}{3}\left(|01\rangle\langle 01|_{BC} + |10\rangle\langle 10|_{BC} + |11\rangle\langle 11|_{BC}\right) \ , \tag{S.36}$$

we find $F(\rho_{ABC}, \mathcal{R}_{B\to BC}(\rho_{AB})) > 0.9829$ and $\sqrt{F(\rho_{ABC}, \mathcal{T}_{B\to BC}(\rho_{AB}))} < 0.9696$, which shows that (S.33) cannot hold since $F\big(\rho_{ABC}, \mathcal{R}_{B\to BC}(\rho_{AB})\big) \leq F(A;C|B)_\rho$.

This does not show that one cannot prove a non-trivial guarantee on the performance of the transpose map relative to the optimal recovery map, but it suggests that such a guarantee would have to be worse than the square root (and actually worse that the fourth root as well using another example), or perhaps it is more naturally expressed using a different distance measure (using similar examples, the trace distance does not seem to be a good candidate, either). We further note that this example does not show that Equation (1.2) is wrong for the transpose map.

References

[BK02] Howard Barnum and Emanuel Knill. Reversing quantum dynamics with near-optimal quantum and classical fidelity. *Journal of Mathematical Physics*, 43(5):2097–2106, 2002.

[FR14] Omar Fawzi and Renato Renner. Quantum conditional mutual information and approximate Markov chains, 2014. arXiv:1410.0664v3.

[FvdG99] Christopher A. Fuchs and Jeroen van de Graaf. Cryptographic distinguishability measures for quantum-mechanical states. *IEEE Transactions on Information Theory*, 45(4):1216 –1227, May 1999.

[Hay01] Masahito Hayashi. Asymptotics of quantum relative entropy from a representation theoretical viewpoint. *Journal of Physics A: Mathematical and General*, 34(16):3413, 2001.

[HP91] Fumio Hiai and D'enes Petz. The proper formula for relative entropy and its asymptotics in quantum probability. *Communications in Mathematical Physics*, 143(1):99–114, 1991.

[NC00] Michael A. Nielsen and Isaac L. Chuang. *Quantum Computation and Quantum Information*. Cambridge University Press, 2000.

[Uhl76] Armin Uhlmann. The "transition probability" in the state space of a *-algebra. *Reports on Mathematical Physics*, 9(2):273 – 279, 1976.

[Win99] Andreas Winter. Coding theorem and strong converse for quantum channels. *IEEE Transactions on Information Theory*, 45(7):2481–2485, Nov 1999.
